# Digital Health Solutions for Type 2 Diabetes and Prediabetes: Systematic Review of Engagement Barriers, Facilitators, and Outcomes

**DOI:** 10.2196/80582

**Published:** 2026-03-12

**Authors:** Ayesha Thanthrige, Nilmini Wickramasinghe

**Affiliations:** 1 School of Computing, Engineering and Mathematical Sciences, La Trobe University, Plenty Road, Bundoora, Victoria, 3086, Australia, 61430601237

**Keywords:** type 2 diabetes mellitus, prediabetes, digital health, artificial intelligence, machine learning, chatbots, engagement, user-centered design

## Abstract

**Background:**

Digital health interventions, including artificial intelligence (AI)-driven solutions, offer promise for type 2 diabetes mellitus (T2DM) and prediabetes management through enhanced self-management, adherence, and personalization. However, engagement challenges and barriers, particularly among young adults and diverse populations, persist. Existing reviews emphasize clinical outcomes while neglecting engagement factors crucial to intervention success. This review highlights engagement barriers and facilitators, offering insights into improving digital health solutions for diabetes management.

**Objective:**

The objective of this systematic literature review is to explore the barriers, facilitators, and outcomes of digital health interventions, focusing on the current state of AI applications while including partial AI and non-AI interventions, for managing and preventing T2DM and prediabetes, to inform the development of user-centered, inclusive digital health interventions for diabetes care. Unlike prior reviews, this review aims to inform the development of user-centered, inclusive digital health interventions for diabetes care, with a focus on engagement across various AI interventions and diverse populations.

**Methods:**

A systematic search of PubMed, Scopus, CINAHL, and additional sources was conducted for studies published between January 2016 and October 2025. Eligibility criteria included English-language, peer-reviewed studies focused on digital health interventions for adults with T2DM or prediabetes, reporting engagement, barriers, facilitators, or outcomes. Data were synthesized narratively using thematic analysis, guided by self-determination theory and user-centered design. Quality appraisal was conducted using Critical Appraisal Skills Program, Mixed Methods Appraisal Tool, and AMSTAR-2 tools.

**Results:**

From the 37 studies (14 quantitative, 3 qualitative, 7 mixed-methods, and 13 reviews), interventions comprised 19 AI-driven (eg, chatbots, ML models, and conversational agent or hybrid), 3 partially AI-driven, and 15 non-AI solutions (eg, apps and lifestyle programs), mostly from the USA (n=15). Key barriers to engagement included inadequate personalization (15/37, 41%), environmental constraints (11/37, 11%), cultural and language mismatches (14/37, 38%), and AI-specific concerns (eg, bias and privacy). Facilitators included personalized feedback (19/37, 51%), cultural tailoring (17/37, 46%), user-friendly design, and peer support. AI-driven interventions demonstrated moderate improvements in clinical outcomes (eg, lowering HbA_1c_, weight loss, and normoglycemia conversion). However, these tools often struggled with keeping users involved and building trust. Non-AI solutions performed similarly but lacked adaptive features.

**Conclusions:**

This review offers novel insights by synthesizing engagement barriers and facilitators across AI and non-AI intervention domains, often neglected in previous studies. It highlights the necessity for testing adaptive, culturally tailored, and user-centered AI interventions to address engagement challenges in T2DM and prediabetes management. Integrating personalization, precision, and value-based care can improve outcomes and scalability. The findings guide the creation of inclusive, AI-driven solutions aligned with self-determination theory and user-centered design principles.

## Introduction

### Background

Diabetes is a critical global public health concern with significant implications for individuals, health care systems, and economies. The International Diabetes Federation reported 537 million adults with diabetes in 2021, projected to increase to 783 million by 2045 [1]. Therefore, global health care spending on diabetes reached approximately US $966 billion in 2021 [2]. Also, type 2 diabetes mellitus (T2DM) complications, including cardiovascular disease, kidney failure, and neuropathy, exacerbate health care costs, particularly in resource-constrained low-middle income countries (LMICs) [3]. Prediabetes, defined by elevated blood glucose levels below the T2DM diagnostic threshold, affected approximately 541 million adults globally in 2021, with significant increases projected by 2030 [1]. Rising obesity rates, sedentary lifestyles, and poor dietary habits worsen the impact of prediabetes [4]. However, prediabetes represents a critical window for intervention to prevent the progression to diabetes, with an estimated 70% lifetime risk of developing T2DM [5] without lifestyle or pharmacological interventions [6].

Digital health technologies have transformed chronic disease management, such as diabetes, by enhancing self-management, improving adherence, and delivering personalized interventions [7]. Furthermore, artificial intelligence (AI)-driven tools, such as chatbots and machine learning models (ML), provide real-time feedback, predictive analytics, and tailored recommendations for better lifestyle choices [8,9]. Hence, these technologies offer scalable solutions to address diabetes and prediabetes management and prevention across diverse populations [10].

While these interventions have potential benefits for individuals, digital health interventions face significant challenges. Recent studies report that high dropout rates and poor sustained engagement reduce the effectiveness of such interventions [11]. Furthermore, AI-specific challenges, such as data availability, cost considerations, AI algorithm performance, bias, and data privacy, emerge as noteworthy barriers hindering the adoption of AI applications and further complicating engagement in diverse populations [12]. Current systematic reviews of digital health interventions for T2DM and prediabetes primarily emphasize clinical outcomes, such as HbA_1c_ reduction and weight loss, which are critical, but limited attention on engagement barriers and facilitators, which are equally important for achieving these clinical outcomes [9,13,14].

### Theoretical Frameworks

Self-determination theory (SDT) provides a robust framework for understanding engagement by emphasizing autonomy (eg, user choice), competence (eg, skill-building), and relatedness (eg, social support) [15]. User-centered design (UCD) principles advocate iterative, user-driven development to ensure usability and alignment with cultural and socioeconomic contexts [16]. Notably, SDT and UCD together guide the development of effective digital health interventions and will allow us to enhance their impact [17,18]. To provide a comprehensive understanding of digital health interventions for T2DM and prediabetes, this review includes both AI-driven and non-AI solutions. Non-AI interventions serve as a baseline to evaluate AI’s added value in addressing engagement barriers and enhancing clinical outcomes, enabling a comparison that informs the design of future AI-driven solutions. This review aims to synthesize engagement barriers, facilitators, and outcomes of digital health interventions for T2DM and prediabetes management across diverse populations, using SDT and UCD frameworks. The specific objectives are (1) to identify barriers to engagement in these interventions, (2) to determine facilitators that enhance engagement across diverse populations, and (3) to evaluate the effectiveness of digital health interventions in achieving clinical outcomes

## Methods

### Study Design and Reporting Guidelines

Systematic literature reviews are usually used to collate all empirical evidence that fits pre-specified eligibility criteria in order to answer a specific research question. It uses explicit, systematic methods that are selected with a view to minimizing bias, thus providing more reliable findings [19]. This systematic literature review was conducted in accordance with the Preferred Reporting Items for Systematic Reviews and Meta-Analyses (PRISMA) guidelines [20]. We used the PRISMA checklist, which enhances the quality, reproducibility, and completeness of the review, enabling researchers to assess the validity of the methods and findings. The review protocol was developed and registered with OSF [21] to ensure methodological transparency.

### Search Strategy

A comprehensive search strategy was developed for Medline (step 1) and refined through consultation with a university librarian using medical subject headings terms (step 2). Multimedia appendix A (Table S1 in Multimedia Appendix 1) represents the search strategy, combining medical subject headings terms and keywords identified with the Population, Intervention, Comparator, and Outcome framework. Then a range of electronic databases was searched, including PubMed, Scopus, and CINAHL supplemented by hand searching and reference lists. (Table S1 in Multimedia Appendix 2) lists databases searched. The search period was restricted to January 2016 through October 2025 to capture recently developed modern digital health interventions, aligning with the rapid evolution of digital health technologies and the post-2015 surge in AI integration (eg, deep learning breakthroughs) [22,23].

The researchers collaborated to determine final papers for inclusion in review through Covidence, the Cochrane Collaboration’s platform for systematic reviews. Inclusion and exclusion criteria for study selection are presented in Textbox 1. In this paper, “Managing” refers to interventions for diagnosed T2DM (eg, self-monitoring, adherence support) [24,25] and “preventing” refers to those for prediabetes to delay onset (eg, lifestyle changes) [26,27]. These categories were applied during full-text review to ensure focus on at-risk or diagnosed adults (

Textbox 1. **Inclusion and exclusion criteria for selecting studies.**

**Inclusion criteria**

- Studies published in English in peer-reviewed journals
- Studies focused on artificial intelligence-driven or digital health interventions (eg, mobile apps, chatbots, SMS text messaging, and wearables) for type 2 diabetes mellitus (T2DM) or prediabetes management or prevention
- Studies included adults aged 18 years to 75 years with T2DM or prediabetes
- Studies reported effectiveness, engagement patterns, barriers, or facilitators
- Studies used quantitative, qualitative, mixed-methods, or review designs
- Studies published between January 2016 and October 2025
**Exclusion criteria**
- Non-English studies and nonpeer-reviewed sources (eg, editorials and abstracts)
- Solutions without a digital component (eg, solely pharmacological)
- Studies targeting only children (<18) or older adults (>75) without broader adult data (to focus on broader adult populations, as those >75 often have unique comorbidities and digital literacy issues that require separate review)
- Studies not reporting engagement, barriers, facilitators, or relevant outcomes
- Studies exclusively on type 1 diabetes, gestational diabetes, or populations without T2DM or prediabetes. (Prediabetes populations are included in this review as they represent a critical window for prevention interventions); only populations without T2DM or prediabetes are excluded
- Studies published before January 2016.

### Study Selection Process

Two reviewers independently screened titles and abstracts followed by full-text review of eligible studies, with disagreements resolved through discussion. Reference lists of included studies and relevant reviews were manually searched for additional studies. The study identification and selection process was documented in a PRISMA flow diagram for transparency. To avoid overlap from the 13 included reviews, primary studies cited within them were cross-checked against our included primaries and only unique insights from reviews were synthesized narratively.

### Quality Assessment

Quality appraisal of included studies was completed by the primary researcher and verified by a second reviewer [28]. Qualitative studies, cohort studies, randomized controlled trials (RCTs), and consensus documents were assessed with the Critical Appraisal Skills Program criteria [29], selecting the appropriate checklist based on study design. Systematic reviews and meta-analyses were appraised using AMSTAR-2 [30]. Mixed-methods and developmental studies were evaluated using the Mixed Methods Appraisal Tool [31]. A scoring system calculated a percentage (number of ‘Yes’ responses divided by total relevant criteria for the study multiplied by 100) with thresholds defined as high (≥80%), moderate (60%‐79%), and low (<60%) [32]. However, studies were not excluded based on quality appraisal. Quality appraisal was primarily conducted by one reviewer with verification by a second, rather than fully independent dual review, potentially introducing minor subjectivity bias.

### Data Extraction

Data were recorded using a standardized form, capturing (1) study characteristics (author, year, country, design, sample size, and demographics), (2) solution details (technology type, duration, theoretical framework, and features), (3) outcomes (categorized as: primary engagement metrics [eg, retention and adherence], secondary behavioral changes [eg, diet and physical activity], clinical [eg, HbA_1c_ and weight]), (4) barriers and facilitators, (5) quality indicators, and (6) qualitative findings on engagement.

### Data Synthesis and Analysis

Due to heterogeneity in study designs, populations, and solutions, a narrative synthesis was used [33]. Quantitative data (eg, dropout rates, HbA_1c_ changes) were summarized descriptively and integrated narratively to support qualitative themes, for example, meta-analytic HbA_1c_ reductions from reviews [13] contextualized thematic barriers such as personalization lacks. A thematic analysis was conducted using an inductive approach to identify barriers and facilitators to engagement, guided by SDT and UCD principles. Themes on barriers and facilitators were derived from reported findings in included studies, using inductive coding, with reviewer interpretation guided by SDT/UCD. The process involved (1) data familiarization, (2) initial coding, (3) theme identification, (4) theme refinement, (5) theme definition, and (6) prevalence quantification. Thematic analysis was conducted by the primary researcher, with themes reviewed and refined by a second researcher. Analysis was supported by NVivo (version 20.7.0, QSR International Pty Ltd), which was used to organize and code data. The PRISMA “qualitative synthesis” refers to the narrative thematic approach due to heterogeneity, not a meta-analysis; no quantitative analysis was feasible.

### Intervention Classification

In this review, “AI-driven interventions” incorporate digital health tools that leverage AI as a core component (eg, ML models and chatbots) or as an enhancement to existing platforms (eg, mobile apps with AI features). “Partially AI-driven interventions” incorporate AI components (eg, ML for tailored messaging) alongside non-AI features (eg, manual data entry), distinguishing them from fully AI-driven interventions and non-AI interventions (eg, SMS text messaging and basic mobile apps). Reviews were classified based on the interventions they evaluate.

### Ethical Considerations

This study is a systematic literature review and did not involve the collection of primary data, enrollment of human participants, or access to identifiable private information. Therefore, according to institutional and national guidelines, this work did not require Institutional Review Board or Research Ethics Board approval. All data analyzed were derived from previously published, peer‑reviewed studies that had obtained their own ethics approval and informed consent as required. No new informed consent was required for this review, as no individual-level or identifiable data were collected, used, or reported. All efforts were made to ensure privacy by using only publicly available summarized findings and by not extracting or presenting any identifiable participant information from the included studies. No participants were recruited for this research and accordingly, no compensation was provided.

## Results

### Study Characteristics

Initial search identified (January 2016 to December 2024) 615 studies and were imported into Covidence. An updated search to October 2025 identified 87 additional records. After screening titles and abstracts, 171 full-text articles were evaluated, with 37 studies [8,9,13,14,34-66] meeting the inclusion criteria for the final narrative synthesis (). The majority of the papers were published in 2024 (n=10). The selected studies encompassed diverse populations, including Chinese Americans, Hispanics, Saudi women, and general adult populations, spanning urban and rural settings. Studies originated from various countries, with the majority from the USA/USA-affiliated studies (n=15), and included China, India, Singapore, and Saudi Arabia (Multimedia Appendix 2). Various study designs were observed, including 7 RCTs [8,37,50,57,61,62,64], 7 systematic reviews and meta-analyses [9,13,14,47,54,56,63], 6 narrative, scoping, or other reviews [38,49,58,59,65,66], 5 observational and cohort studies [36,51-53,55], 3 qualitative studies [44,45,60], and 7 mixed-methods studies [39-43,46,48].

**Figure 1.**
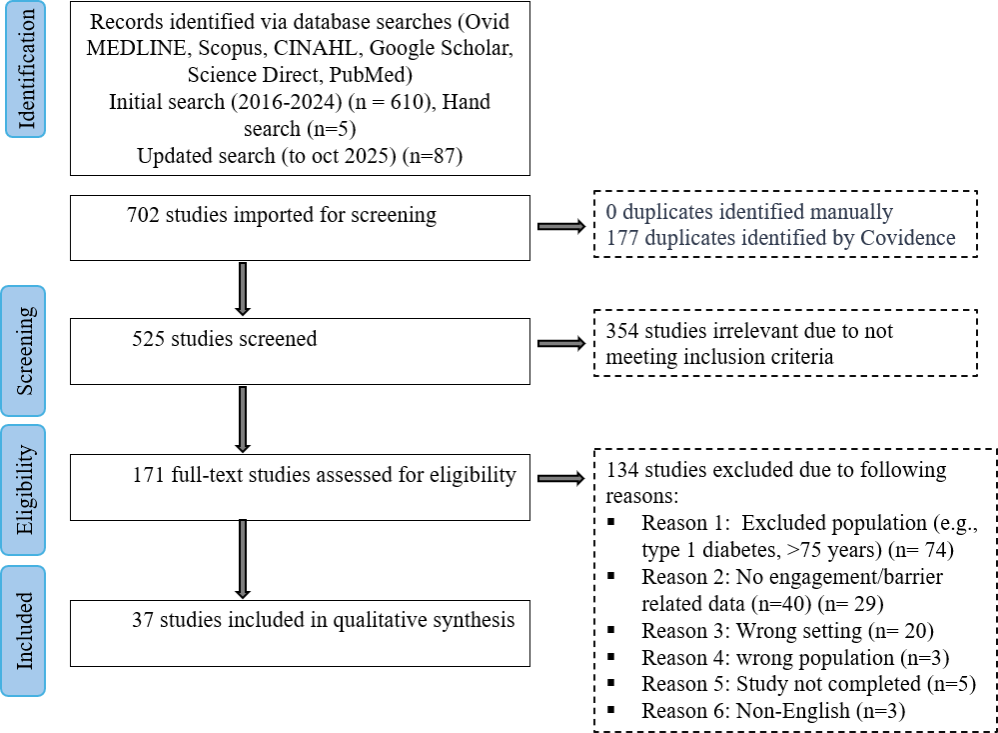
Study identification and selection process. PRISMA (Preferred Reporting Items for Systematic Reviews and Meta-Analyses) flow diagram.

Among the 37 studies [8,9,13,14,34-66], 13 are fully AI-based [8,34,35,37,39,40,44-46,50-52,60]: 7 use chatbots, large language models, or conversational agents [8,34,39,40,44-46], 3 use ML models [37,51,60], 2 involve AI-led lifestyle interventions or digital twins [50,52], and 1 uses voice or image recognition [35]. These studies fully leverage technologies, such as chatbots, ML models (many using extreme gradient boosting), conversational agents, and voice or image recognition systems. In total, 8 studies [36,42,43,48,53,57,61,64] are non-AI, consisting of 2 mobile apps, 4 lifestyle programs, 1 SMS text messaging-based intervention, and 1 gamified mHealth application without AI components, relying instead on traditional methods such as lifestyle interventions and conventional digital health tools. Three studies [41,55,62] are partially AI-based, combining AI features with human-led or manual components, a mobile app with tailored messaging and a provider portal, an AI-powered app with automated cues plus dietitian chat, and automated insulin titration systems. The remaining 13 studies[9,13,14,38,47,49,54,56,58,59,63,65,66] are reviews, of which 6 focus on AI applications and 7 address non-AI or broader digital health interventions. Figure 1 details the PRISMA flowchart summary.

### Quality Assessment

Table S1 reports the quality appraisal scores for the 37 [8,9,13,14,34-66] studies, assessed using standardized tools, such as Critical Appraisal Skills Program, Mixed Methods Appraisal Tool, and AMSTAR-2, reflecting rigorous evaluation of methodological quality, with scores ranging from 75% to 100%. No studies were excluded based on quality, underlining the overall rigor of the included research.

### Thematic Analysis

Thematic analysis was guided by self-determination theory and UCD principles. Table 1 summarizes themes, subthemes, and findings of digital health interventions.

**Table 1.** Themes, subthemes, and findings for digital health interventions in type 2 diabetes mellitus and prediabetes.

Theme	Summary of findings in studies
Barriers to engagement	Factors affecting user engagement and sustainable use of digital health interventions for T2DM^a^ and prediabetes, impacting SDT^b^ elements autonomy, competence, and relatedness. High dropout rates (6.4%‐35.5% across 14/37 studies) served as a quantifiable indicator of low engagement, primarily due to low-risk perception, inadequate personalization, and lack of motivation [34].
Inadequate Personalization	Found in 15/37 studies (41%), reducing motivation due to generic content or lack of cultural sensitivity includes generic meal plans in ChatGPT responses [34], limited Chinese-specific dietary advice [35], nonindividualized calorie, targets in a formula diet RCT^c^ [64], and poor demographic adaptation in ML models [51,60].
Low risk perception	Reported in 4/37 studies (11%), low perceived susceptibility reduced relevance and early adherence particularly among younger adults as users saw interventions as irrelevant [36,37].
Environmental constraints	Present in 11/37 studies (30%), regional health care system variations [38], inadequate access to advanced devices in low-income countries [39], limited smartphone/internet access [9,40], affordability challenges and poor digital literacy [41,42], and infrastructural issues in low-resource settings [57] led to reduced adoption and increased dropout, especially in rural populations [26].
Cultural and language barriers	Found in 14/37 studies (38%), include English-only interfaces [43,44], limited non-English conversational agents [45], limited localization [40] cultural mismatches in content [8,41,42] and culturally insensitive dietary recommendations [34,35] reduced engagement.
AI^d^-specific barriers	Reported in 5/37 studies (14%), include potential for fabricated information [44], limited intent recognition, nonadaptive rules-based systems [8], and inconsistent responses [46], compromising accuracy and personalization in AI-driven interventions.
Socioeconomic barriers	identified in 13/37 studies (35%) include limited smartphone access [47], low digital literacy [42], and restricted access to care [58], hindering adoption and effectiveness in resource-constrained populations.
Facilitators of engagement	Factors enhancing user engagement and adherence, supporting autonomy, competence, and relatedness (SDT) and aligning with UCD^e^ principles.
Cultural and linguistic tailoring	Reported in 17/37 studies (46%), enhanced relatedness, by culturally adapted carbohydrate tracking [43], multilingual support with local accents [45], Persian food databases [41], Hispanic-focused soccer programs [36], Arabic WhatsApp peer groups [61], Chinese American web-based DPPs^f^ [48] and Chinese-specific dietary recommendations [35].
Personalized and adaptive feedback	Found in 19/37 studies (51%), improved motivation through AI-driven adaptivity or gamification, includes use of personalized AI interactions and conversational empathy [9,14], AI-led DPP [50], personalized, adaptive or AI-powered feedback [35,51,52] and non-AI personalized communication or program choice [53].
User-friendly design	Identified in 5/37 studies (14%), include simple interfaces with no login [51], voice-based interactions [8], automated reminders [9], user-friendly WeChat mini-program [35] and multi-platform access [40], enhancing usability and engagement.
Peer support and social features	Observed in 9/37 studies (24%), include community-driven co-production [60], social support via WhatsApp [36], private Facebook groups [48], family involvement in culturally tailored DPPs [36], buddy systems in hybrid apps [37], and community features in apps [54], enhancing engagement.
Telemonitoring and real-time feedback	Found in 10/37 studies (27%), include continuous glucose monitoring and Fitbit integration [51], real-time data portals [8], real-time monitoring [67], automated alerts through AI systems [50] and cloud-based provider portals [41], built competence and accountability via real-time tracking.
Clinical and behavioral outcomes	Measurable impacts of digital health interventions on health outcomes and user behaviors, critical for evaluating effectiveness.
HbA^g^_1c_ reduction	HbA_1c_ reduction outcomes in diabetes management include a 0.3% decrease with chatbots [9], 0.39% with mobile apps [47], 1.8% with digital twin tech [52] and 1.6% with telehealth interventions [56], improving glycemic control.
Weight loss	Weight loss outcomes in diabetes management include 1.3 kg with chatbots [14], 10.6% with app engagement [55], 7.3% at 6 months [53], up to 6.5 kg in multi-strategy DHIs^h^ [56] and 5.9 kg with lifestyle interventions [64].
Normoglycemia conversion	Normoglycemia conversion outcomes in diabetes management show a 50% conversion rate with a low-carbohydrate diet intervention versus 31% with lifestyle alone [64], indicating enhanced reversal of prediabetes through technology-supported dietary strategies.
Improved physical activity	Improved physical activity outcomes in diabetes management include increased step-goal achievement with chatbots [14], enhanced VO2^i^ max and agility [36], and improved activity with app-based tracking [47].
Enhanced dietary management	Enhanced dietary management outcomes in diabetes management include improved diet with chatbots [14], 96.43% acceptable ketogenic diet responses [35], and better energy intake with lifestyle interventions [68].
Increased user engagement and adherence	Increased engagement/adherence as primary outcomes, include AI nutrition system with 96.43% valid ketogenic diet recommendations [35], culturally adapted meal adherence [43,48], improved food intake and energy management in mobile and lifestyle programs [47], 89.3% data logging with voice-based AI [8], 65% program completion [53], high chatbot acceptance [9], and 85% retention [48].

a T2DM: type 2 diabetes mellitus.

b SDT: self-determination theory.

c RCT: randomized controlled trial.

d AI: artificial intelligence.

e UCD: User-centered design.

f DPP: diabetes prevention program.

g HbA_1c_: hemoglobin A1c.

h DHI: digital health intervention.

i VO2: volume of oxygen.

### Barriers to Engagement

The thematic analysis identified key barriers such as low-risk perception, inadequate personalization, environmental constraints, cultural or language mismatches, AI-specific concerns (eg, bias, privacy), and socioeconomic barriers. Dropout rates (6.4%‐35.5% across 14 studies [8,34,36,48-53,55,57,61,62,64]) served as an indicator of low engagement, primarily due to underlying causes such as inadequate personalization and motivational lacks, rather than a standalone theme. For example, declining app usage linked to repetitive content [51] and declining motivation over time [34,36], suggesting gamification as a solution, reinforced by generic features in non-AI reviews [41,56].

Declining use over time was frequently attributed to repetitive content and inadequate personalization, both of which reduced users’ sense of competence and motivation. For instance [55], reported declining nBuddy app usage, while [35] noted inconsistent AI-driven dietary advice due to limited personalization. Additional studies, such as [49] and [36], highlighted similar challenges with maintaining user motivation over time [34], noted poor sustained engagement from lack of follow-up, and [36] reported declining physical activity post-intervention. Studies suggested gamification, social incentives, and adaptive feedback as remedies to sustain interest.

Inadequate personalization, identified as a critical barrier in 15 [34,35,37,39-41,46,47,50-52,55,57,60,64] of 37 studies[8,9,13,14,34-66] (41%), weakened users’ sense of competence and relevance. Static feedback, generic goal-setting, and nonadaptive content led to disengagement. Examples include generic dietary advice in nontailored chatbots [40], poor demographic adaptation in ML models [39,51], and limited personalization in traditional programs [47,69]. Although AI-driven interventions with adaptive algorithms [35,52] demonstrated improved engagement and outcomes, they still exhibited constraints in fine-grained individualization. Several studies emphasized the importance of tailoring content to user preferences, cultural context, and progress level. Examples include generic meal plans in ChatGPT (OpenAI) responses [34], limited Chinese-specific dietary advice [35], and nonindividualized calorie, targets in a formula diet RCT [64].

Low-risk perception, identified in 4 studies, [34,36,37,49] especially among younger adults, undermined intrinsic motivation and autonomy, leading to disengagement. Participants often viewed interventions as irrelevant to their immediate health needs. Several lifestyle-based, non-AI studies [34,36,49] reported decreased participation due to perceived low personal diabetes risk and lack of urgency.

Environmental constraints, noted in 11 [9,26,38-42,48,53,57,61] out of 37 studies [8,9,13,14,34-66] (30%), included socioeconomic barriers, such as limited smartphone access, and technical challenges, such as complex interfaces, affordability, and accessibility. For instance [42], highlighted technology unfamiliarity among older adults, and [48] reported navigation difficulties. Other studies, including [53] and [61], emphasized socioeconomic barriers, such as transportation or resource limitations, which further hindered engagement.

Cultural/language barriers, in 14 [8,34-36,40-45,47,48,53,61] out of 37 studies[8,9,13,14,34-66] (38%), reduced engagement in diverse populations due to noninclusive content. For example [43], reported an English-only DiaFriend app, and [40] noted an Italian-only AIDA chatbot, and [68] reported high dropout rates (not quantified) in an Arabic-only WhatsApp program, while culturally tailored interventions showed better retention, such as [48] with 15% dropout in a Chinese American web-based diabetes prevention program (DPP). These issues hinder accessibility and personalization, particularly for non-English-speaking and culturally diverse populations, reducing intervention effectiveness.

AI-specific barriers, in 5 [8,40,44-46] out of 37 studies [8,9,13,14,34-66] (14%) included potential for fabricated information [44], constrained input and miscommunication risks [45], limited intent recognition with 9% misclassification [40], and nonadaptive rules-based systems [45], compromising trust, accuracy, and personalization in AI-driven interventions. Socioeconomic barriers, in 13 [36,37,41-43,47,48,53,55,57,58,61,64] out of 37 studies [8,9,13,14,34-66] (35%), involved limited smartphone access [57], resource constraints and access issues [58], limited digital literacy and technology unfamiliarity among older adults [42], smartphone literacy requirements [41], and cost of devices and data.

### Facilitators of Engagement

Engagement facilitators were identified in studies reporting high retention or engagement. Cultural tailoring, in 17 [9,14,35,36,40-43,45,48,51,54,55,58,60,61,66] out of 37 studies [8,9,13,14,34-66] (46%), enhanced relatedness through relevant content, as seen in [43] with the culturally tailored app and [36] with a Hispanic-focused program, Portuguese American carbohydrate tracking [43] and [48] with Chinese American web-based DPP (85% retention).

Personalized feedback, in 19 studies [8,9,14,34,35,37,40-42,44-46,50-53,55,57,62] (51%), sustained motivation via adaptive features or gamification. Examples include [8] with voice-based conversational AI achieving 82.9% adherence and 89.3% data logging [50], with AI-powered adaptive interventions showing 93.4% initiation and 63.9% completion rates, and [52] with digital twin technology showing 50.7% diabetes remission rates. User-friendly design, in 5 studies [8,9,35,40,43] (14%), improved accessibility with intuitive interfaces [43]. featured simple interfaces with no login requirements [8], used voice-based interactions for ease of use [40], provided multi-platform access (Telegram [Telegram Messenger LLP], website, Alexa [Amazon]), and [35] offered a user-friendly WeChat (Tencent) mini-program. Peer support, in 9 studies [36,37,48,54,55,58,60,61,66](24%), fostered community engagement, as evidenced by [48] with a Facebook (Meta) group and [61] with WhatsApp support groups [36], with social support via WhatsApp and family involvement, and [37] with a buddy system, enhancing sustained engagement. Telemonitoring and real-time feedback (10 out of 37 [27%]) enhanced engagement via continuous glucose monitors (CGM) or Fitbit integration [51], telemonitoring with scales and pedometers [45], and cloud portals [41], enabling timely intervention adjustments and competence-building.

### Clinical and Behavioral Outcomes

Thematic analysis, guided by SDT and UCD, reveals that AI-based interventions for T2D self-management foster autonomy and competence, yielding significant clinical behavioral outcomes, including HbA_1c_ reductions (0.19%‐1.8%), improved diet and physical activity adherence, and weight loss (0.8%‐10.6%) [9,47,55]. Culturally tailored tools and voice-based AI enhance relatedness and engagement, supporting glycemic control [8,43]. Non-AI interventions, such as lifestyle programs, contribute similarly but lack adaptive personalization, emphasizing AI’s potential to address SDT-driven motivation gaps [36].

### The Application of AI in Diabetes Care

**Figure 2.**
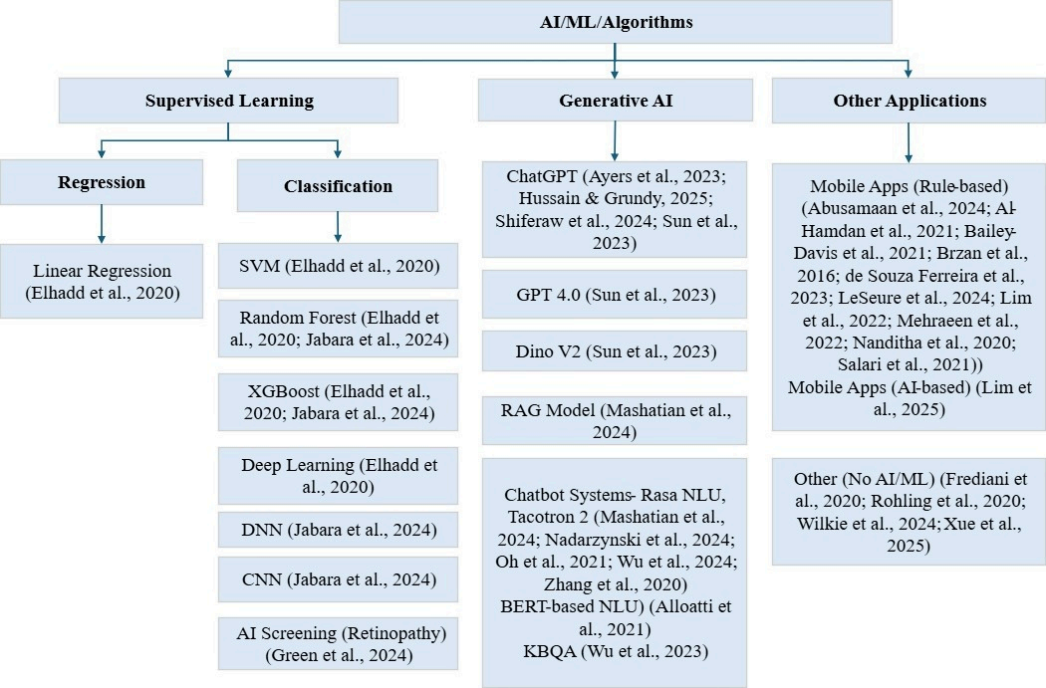
Classification of artificial intelligence, machine learning, and algorithms in diabetes/prediabetes management. BERT representations from transformers. AI: artificial intelligence; BERT: Bidirectional Encoder; CNN: convolutional neural network; DNN: deep neural network; GPT-4: generative pre-trained transformer 4; KBOA: knowledge-based question answering; NLU: natural language understanding; RAG: retrieval-augmented generation; SVM: support vector machine; XGBoost: extreme gradient boosting [9,13,14,34-36,39-44,46,47,49,51,53-55,57-61,67,69,70].

The application of AI in diabetes care, as evidenced by 19 AI-based studies [8,9,14,34,35,37-40,44-46,49-52,59,60,65] among the 37 analyzed, has shown significant potential to enhance engagement and clinical outcomes through advanced methodologies (Figure 2). Quantitative results from a small subset of trials demonstrated moderate-to-high engagement (63.9%‐93.4%) and relatively low dropout (6.6%‐17.9%) compared with non-AI interventions [8,50,52]. For instance, an AI-powered digital twin intervention achieved a 1.8% reduction in HbA_1c_ and 4.8 kg weight loss over one year with 6.6% attrition [52], while a voice-based AI assistant for insulin titration reported 82.9% adherence and faster dose optimization relative to standard care [8]. ML models such as extreme gradient boosting achieved high accuracy in predicting glucose variability (*R*²=0.837) [51] and enabled precise screening and complication detection using digital biomarkers from sensors, such as electrocardiograms and photoplethysmography (eg, IDx-DR, an AI-based diabetic retinopathy screening tool: 87.2% sensitivity, 90.7% specificity) [59]. Large language models (LLMs), including ChatGPT and GPT-4, delivered tailored dietary advice, with [35] reporting 74.5% accuracy on the Chinese Registered Dietitian Exam and 96.43% of ketogenic diet responses rated acceptable or excellent, though limited by inconsistent recommendations for Chinese-specific foods. Conversational agents, such as AIDA [40], reached approximately 4000 unique users with 91% intent recognition accuracy, while AMANDA [45] offered multilingual support with a Singaporean-accented text-to-speech feature, achieving high usability (System Usability Scale score=80.625) and positive user experience ratings (Mean Opinion Scores: 4.07 for naturalness, 3.98 for accent uniqueness, 3.88 for clarity).

Hybrid approaches such as the retriever-augmented generation model provided 98% accuracy in patient education for diabetes and diabetic foot care [39]. AI-driven interventions, such as the Sweetch app [67], used just-in-time adaptive interventions, improving adherence (82.9%) [8]. However, challenges included AI-specific barriers in 16% of studies, such as algorithmic bias [38,46], privacy concerns [59], and inconsistent responses [34], and alongside inadequate personalization (38% of studies) often due to generic content or lack of adaptive features [9,49,58]. Culturally tailored solutions, such as the DiaFriend app for Portuguese Americans [43] and AI-HEALS for Chinese patients [70], mitigated some barriers but faced limitations such as incomplete backends or language restrictions. These findings highlight AI’s transformative potential in diabetes management while emphasizing the need for future large-scale, comparative, and longitudinal studies to determine their real-world effectiveness, cost-efficiency, and equity impact across diverse populations. Addressing biases, privacy, cultural adaptation, and sustained engagement challenges to optimize future implementations. Table 2 summarizes the comparison of AI-driven versus non-AI interventions.

**Table 2.** Comparison of artificial intelligence-driven versus non-artificial intelligence interventions.

Dimension	AI^a^	Partial-AI	Non-AI
Dropout range	6.6%-30% (n=3) [8,49,52].	6.4% (n=1) [55].	14.4%-35.5% (n=4) [53,57,61,64].
Engagement range (primary outcome)	63.9%-100% (n=4) [8,9,14,50].	Not mentioned.	65%-92% completion or retention (n=2) [48,53].
HbA^b^_1c_ reduction range	0.2%‐1.8% (n=3) [9,50,52].	1%‐1.2% (n=2) [55,62].	0.19%‐1.6% (n=8) [13,37,47,53,56,58,61,63].
Barriers	Inadequate personalization [34,35,44,46,51], low engagement and adherence [34,44-46,50-52], technical issues and connectivity [35,44,51], short duration and follow-up [35,44,50-52], cultural and language issues [44], and poorly defined AI taxonomy [52].	Inadequate personalization [41,55,62], dropout and engagement issues [55,62], limited interactivity [55], cultural or language barriers [41,55], and limited clinician time [62].	Inadequate personalization [36,42,43,48,53,57,61,64], dropout or engagement issues [36,42,43,48,53,57,61,64], limited interactivity [42,43,48,53], and cultural or socioeconomic barriers [42,43,48,53,57,61]
Facilitators	Personalized feedback and adaptive algorithms [34,35,44,46,50-52], behavioral model integration [37,50,52], remote monitoring and provider feedback [44,50-52], user-friendly design [34,35,44,46,51], culturally tailored content [44], cloud integration for provider access [51,52], structured follow-up improving adherence [8,50,52], scalable interventions [39,50,52], integration of AI and human support [52], and emphasis on scalability and precision [52]	Tailored feedback and reminders [41,55,62], automated tracking features [41,55,62], behavioral frameworks (CBT^c^, goal setting) [55,62], provider monitoring [55], educational and motivational support [41,55], cultural adaptation [41,55,62], and local food databases [55,62], real-time communication [55].	Educational content [42,43,48,53,57,61,64], human coaching and social support [42,43,48,53,58,61], culturally appropriate design [42,43,48,53,61], motivational reinforcement [48,53,58,61], health care professional guidance [42,48,53,58,61], simplicity and accessibility [48,53,58,61], integration with health care systems [42,48,53,58,61], supportive follow-up [48,53,58,61], structured educational design [13,63], emphasis on usability and accessibility [13,63], incorporation of behavioral science [13], positive patient–provider communication [13,63], and evidence synthesis improving generalizability [63].
Clinical outcomes	HbA_1c_ reduction [50,52], weight loss [50], improved self-management and adherence [44,50,52], improved patient satisfaction [35,44], no increase in hypoglycemia [52], scalability potential [39,50,52], positive usability outcomes [35,44], and clear trends toward improved glycemic control [52]	Improved glycemic control [41,55,62], weight loss [41,55], better engagement with mixed-mode support [55,62], improved knowledge and self-efficacy [55], feasibility demonstrated [55], and moderate-to-high user satisfaction [55].	Improved self-management [42,43,48,53,57,61,64], enhanced knowledge and motivation [42,43,48,53,57,61,64], weight reduction [48,53,61,64], HbA_1c_ reduction [48,53,61,64], improved patient confidence [42,43,48,53,61], high acceptability [58], positive behavioral outcomes [42,43]. Consistent improvement in self-care outcomes [13,63] reinforced the need for personalized interventions [13] and positive health literacy and behavioral impact [63].

a AI: artificial intelligence.

b HbA_1c_: hemoglobin A1c.

c CBT: cognitive behavioral therapy.

### Engagement Across Diverse Populations

Across the 37 [8,9,13,14,34-66] studies, only 8 explicitly targeted culturally or linguistically diverse populations, Portuguese Americans [43], Iranian adults [41,42], Singaporean users [45,55], Arab women [61], Hispanic men [36], and Chinese Americans [48] (Table 3). These studies collectively highlight how cultural adaptation enhances engagement and usability, though most lacked long-term quantitative evaluation. High engagement and retention were most evident in culturally grounded, community-based interventions. The Facebook-delivered DPP for Chinese Americans achieved 85% retention at one year [48], while the soccer-based Latino men’s program retained 65% at 24 weeks [36]. Among Arabic-speaking women in Saudi Arabia, retention reached 100% despite cultural restrictions [61]. In contrast, prototype or design-phase studies in Iran and the US [41-43] did not measure engagement but emphasized usability and localized content. AI-enabled programs from Singapore [45,55] demonstrated high usability (system usability scale 80.6) and low dropout (6.4%), reflecting benefits of linguistic personalization and local food databases in digitally literate populations. Common barriers included low digital literacy, gender or mobility restrictions, and limited multilingual functionality, while facilitators centered on language adaptation, cultural familiarity, and social support. Overall, cultural adaptation consistently improved acceptability, but few studies measured sustained engagement, an important focus for future research.

**Table 3.** Population diversity and engagement metrics across studies.

Population and study IDs	Country	AI^a^ type	Cultural adaptation	Engagement or retention	Key findings	Main facilitators (F) and barriers (B)
Portuguese Americans [43]	USA	Non-AI	Portuguese food and visuals	Not measured	Prototype only and expected to improve adherence	Simple interface (F) and English-only backend (B)
Iranian [41,42]	Iran	AI or Partial-AI	Persian language, food DB^b^, and TTM^c^ tailoring	Usability only (short-term)	Positive clarity or usability and no outcome data	Localized design (F) and low digital literacy (B)
Singaporean [45,55]	Singapore	AI or Partial-AI	Multilingual TTS^d^ and local food DB	SUS^e^ 80.6, 6.4% dropout	High usability: engagement linked to weight loss	Personalization (F) and manual logging burden (B)
Arab women [68]	Saudi Arabia	Non-AI	Arabic-language and gender norms	100% retention	HbA^f^_1c_ reduction (*P<.001*), feasible WhatsApp delivery	Cultural tailoring (F) and mobility limits (B)
Hispanic men [36]	USA	Non-AI	Bilingual coaches and soccer	65% retention	Improved fitness, motivation, and social bonding	Peer support (F) and time and work barriers (B)
Chinese Americans [48]	USA	Non-AI	Bilingual modules	85% retention (1y)	Improved satisfaction and 2.3% weight loss	Coach support (F) and low online literacy (B)

a AI: artificial intelligence.

b DB: dietary behavior.

c TTM: transtheoretical model.

d TTS: transtheoretical stage.

e SUS: system usability scale.

f HbA_1c_: hemoglobin A1c.

## Discussion

### Overview

This systematic review synthesized engagement barriers, facilitators, and outcomes across AI-driven, partially AI, and non-AI digital health interventions for T2DM and prediabetes. By applying SDT and UCD as interpretive frameworks, this review extends prior work that has predominantly focused on clinical outcomes [27,48]. Our findings show that the most prevalent barriers to sustained user engagement were inadequate personalization, cultural or language mismatches, socioeconomic constraints, and, in AI tools, specific concerns about bias and privacy, while the strongest facilitators were personalized and adaptive feedback and cultural tailoring. These factors, through their influence on autonomy, competence, and relatedness and usability, appear to be the primary drivers of behavioral and clinical change in digital diabetes interventions.

### Main Findings

Across the 3 7[8,9,13,14,34-66] included studies, digital health interventions demonstrated meaningful improvements in glycemic control, dietary behaviors, and physical activity, though sustained engagement remained a critical challenge. Inadequate personalization emerged as one of the most prevalent barriers, undermining SDT’s principles of autonomy and competence and contributing to dropout. AI-based tools generally outperformed traditional digital programs when adaptivity, real-time monitoring, and tailored feedback were effectively implemented. For instance, AI-supported insulin titration and predictive analytics achieved faster glycemic improvements than standard care [5]. Interventions incorporating cultural tailoring, social support, and simplified interfaces consistently demonstrated higher engagement and completion rates. These findings emphasize that algorithmic sophistication alone is insufficient; meaningful engagement depends on how well digital systems address users’ psychological needs, contextual realities, and cultural identities.

### Engagement in Diverse Populations

Engagement varied substantially across demographic, cultural, and socioeconomic groups. Young adults frequently demonstrated low perceived risk and weaker intrinsic motivation to sustain engagement, while older adults faced usability challenges and digital literacy barriers. Cultural mismatch, reported in nearly 40% of studies, led to reduced trust and relevance, particularly among minority groups.

Socioeconomic constraints such as limited smartphone access, high data costs, or inconsistent internet connectivity were especially apparent in LMIC settings. Interventions that provided multilingual content, culturally relevant dietary databases, or low-bandwidth delivery (eg, SMS text messaging-based chatbots) showed higher acceptability and engagement. These results emphasize the importance of context-aware and culturally grounded design practices when delivering digital diabetes interventions at scale.

### Barriers to Engagement and Trust in AI

Several AI-specific barriers affected user trust and engagement. Algorithmic bias, arising from nonrepresentative training datasets, resulted in inaccurate risk predictions or poorly matched recommendations, particularly among ethnically diverse populations [42]. Privacy concerns were common, especially in cloud-based systems, with several users expressing discomfort about data security or opaque data handling processes [65]. Such concerns directly undermine SDT’s relatedness and competence needs by diminishing the sense of transparency and credibility.

LLM-based or rule-based conversational agents occasionally produced generic, repetitive, or incorrect responses, which weakened trust and reduced perceived intervention quality. In contrast, systems that provided transparent rationales (eg, via Explainable AI), culturally adapted messaging, or adaptive learning mechanisms fostered significantly stronger engagement.

These insights highlight that strong technical performance does not guarantee user trust; trust must be actively cultivated through transparency, reliability, cultural sensitivity, and robust data governance.

### Clinical and Behavioral Outcomes

Both AI-driven and non-AI digital interventions yielded positive clinical and behavioral outcomes. AI-enabled programs leveraging CGM, predictive modeling, or digital twins produced some of the largest HbA_1c_ reductions and behavioral improvements observed within the included studies [23,27]. Traditional digital interventions, such as structured online modules or SMS text messaging coaching, offered modest but consistent improvements in diet, physical activity, and self-management.

However, across all intervention types, the effectiveness of clinical outcomes was closely linked to user engagement. Interventions that successfully supported autonomy (eg, personalized goal setting), competence (eg, timely feedback), and relatedness (eg, social support) achieved higher adherence and more sustained improvements. These findings reinforce that engagement is not merely a process measure but a core determinant of intervention effectiveness.

### Limitations

This review provides insights into digital health interventions for T2DM and prediabetes, but several limitations should be acknowledged. The Population, Intervention, Comparator, and Outcome-based search strategy may have missed studies using nonstandardized AI or digital health terms, and the English-only focus excluded relevant non-English or gray literature, potentially limiting the generalizability of findings to global populations. The review did not fully address informatics challenges, which are critical for scalability. Additionally, findings were not stratified by intervention type (AI-driven, partially AI-driven, vs non-AI) or by study setting and population due to limited quantitative data availability, which may restrict understanding of differences in engagement patterns and barriers across these subgroups.

The exclusion of studies targeting only children or older adults without broader adult data restricts insights into these populations, as older adults often have unique comorbidities and digital literacy challenges requiring separate evaluation. Methodological limitations of included studies, such as small sample sizes and heterogeneous designs, may affect generalizability. Quality appraisal was primarily conducted by a single reviewer and verified by a second, rather than through fully independent dual review, which may introduce subjective bias. Additionally, the inclusion of prior systematic reviews alongside primary studies introduces potential evidential overlap. We mitigated this by extracting only novel insights from the reviews; however, a small amount of overlap in the evidence is possible. This review provides a robust foundation for understanding engagement barriers and facilitators in digital health interventions for adults with T2DM and prediabetes.

### Future Directions

The results from this review point to several important areas for future research. Longitudinal trials are needed to assess the long-term impacts of AI-driven interventions, particularly in terms of sustained engagement, clinical outcomes, and cost-effectiveness across diverse populations. Future studies should also explore AI-enabled solutions that integrate real-time data, such as CGM and wearables, to offer more precise, tailored interventions that enhance motivation and adherence.

Furthermore, inclusive research is needed to explore the effectiveness of digital health interventions in LMICs and underserved populations, where cultural sensitivity and accessibility are critical. Additionally, interoperability between AI tools and existing health care systems, such as electronic health records, must be addressed to facilitate seamless data sharing and personalized care. Research should focus on the integration of AI with established health care platforms, enabling a holistic approach to patient management.

Additionally, future studies should explore low-cost, offline solutions such as SMS text messaging-based multilingual chatbots, which can help bridge digital health access gaps in LMICs. Interdisciplinary collaborations between health informatics, behavioral science, and policy experts will be crucial for evaluating the scalability and ethics of these solutions globally. Ethical considerations, including data privacy, consent, and equitable access, must also be central to future research agendas.

### Conclusions

This systematic review provides important insights into the design and implementation of digital health interventions for T2DM and prediabetes management, emphasizing the need for adaptive, inclusive, and user-centered solutions. Both AI-driven and non-AI interventions have shown promise in improving clinical outcomes and engagement, though each faces unique challenges. The integration of SDT and UCD principles, alongside advances in AI technology, can lead to more personalized and equitable solutions for diabetes care. Future research must prioritize diverse populations, cultural tailoring, and advanced informatics techniques to address current barriers and optimize the potential of digital health interventions in global diabetes prevention.

## Supplementary material

10.2196/80582 Multimedia Appendix 1 Detailed search strategy.

10.2196/80582 Multimedia Appendix 2 Detailed study characteristics and findings.

10.2196/80582 Checklist 1 PRISMA checklist.

## Abbreviations

AI: artificial intelligence
CGM: continuous glucose monitors
DPP: diabetes prevention program
LMIC: low-middle income countries
PRISMA: Preferred Reporting Items for Systematic Reviews and Meta-Analyses
RCT: randomized controlled trial
SDT: self-determination theory
T2DM: type 2 diabetes mellitus
UCD: user-centered design

## References

[1] Diabetes facts and figures. International Diabetes Federation.

[2] Sun H, Saeedi P, Karuranga S (2022). IDF diabetes atlas: global, regional and country-level diabetes prevalence estimates for 2021 and projections for 2045. Diabetes Res Clin Pract.

[3] Godman B, Basu D, Pillay Y (2020). Review of ongoing activities and challenges to improve the care of patients with type 2 diabetes across africa and the implications for the future. Front Pharmacol.

[4] Sami W, Ansari T, Butt NS, Hamid MRA (2017). Effect of diet on type 2 diabetes mellitus: a review. Int J Health Sci (Qassim).

[5] Reed J, Bain S, Kanamarlapudi V (2021). A review of current trends with type 2 diabetes epidemiology, aetiology, pathogenesis, treatments and future perspectives. Diabetes Metab Syndr Obes.

[6] Sui CF, Ming LC, Soh YC, Ng CH, Al-Worafi YM, Hussain Z (2025). Systematic review protocol for effectiveness and cost-effectiveness of non-surgical interventions to prevent diabetes progression in adults with prediabetes. BMJ Open.

[7] Gardner DSL, Saboo B, Kesavadev J (2025). Digital health technology in diabetes management in the Asia-Pacific region: a narrative review of the current scenario and future outlook. Diabetes Ther.

[8] Nayak A, Vakili S, Nayak K (2023). Use of voice-based conversational artificial intelligence for basal insulin prescription management among patients with type 2 diabetes: a randomized clinical trial. JAMA Netw Open.

[9] Wu Y, Zhang J, Ge P (2024). Application of chatbots to help patients self-manage diabetes: systematic review and meta-analysis. J Med Internet Res.

[10] Aggarwal A, Tam CC, Wu D, Li X, Qiao S (2023). Artificial intelligence-based chatbots for promoting health behavioral changes: systematic review. J Med Internet Res.

[11] Meyerowitz-Katz G, Ravi S, Arnolda L, Feng X, Maberly G, Astell-Burt T (2020). Rates of attrition and dropout in app-based interventions for chronic disease: systematic review and meta-analysis. J Med Internet Res.

[12] Mbanugo OJ (2025). AI-enhanced telemedicine: a common-sense approach to chronic disease management and a tool to bridging the gap in healthcare disparities. Int J Res Publ Rev.

[13] Xue H, Zhang L, Shi Y (2025). The effectiveness of digital health intervention on glycemic control and physical activity in patients with type 2 diabetes: a systematic review and meta-analysis. Front Digit Health.

[14] Oh YJ, Zhang J, Fang ML, Fukuoka Y (2021). A systematic review of artificial intelligence chatbots for promoting physical activity, healthy diet, and weight loss. Int J Behav Nutr Phys Act.

[15] Ryan RM, Deci EL (2000). Self-determination theory and the facilitation of intrinsic motivation, social development, and well-being. American Psychologist.

[16] Norman DA, Draper SW (1986). User Centered System Design: New Perspectives on Human-Computer Interaction.

[17] Prince G, Rees Lewis D, Pollack T (2024). Employing user-centered design and education sciences to inform training of diabetes survival skills. J Clin Transl Endocrinol.

[18] Mathiesen AS, Zoffmann V, Lindschou J (2023). Self-determination theory interventions versus usual care in people with diabetes: a systematic review with meta-analysis and trial sequential analysis. Syst Rev.

[19] (2019). Cochrane Handbook for Systematic Reviews of Interventions.

[20] Page MJ, McKenzie JE, Bossuyt PM (2021). The PRISMA 2020 statement: an updated guideline for reporting systematic reviews. BMJ.

[21] Optimizing engagement in digital health interventions for diabetes and prediabetes: a systematic review. OSF.

[22] Razavi S (2021). Deep learning, explained: Fundamentals, explainability, and bridgeability to process-based modelling. Environ Model Softw.

[23] Bahadori S, Buckle P, Soukup Ascensao T, Ghafur S, Kierkegaard P (2025). Evolving digital health technologies: aligning with and enhancing the national institute for health and care excellence evidence standards framework. JMIR Mhealth Uhealth.

[24] Carpenter R, DiChiacchio T, Barker K (2019). Interventions for self-management of type 2 diabetes: an integrative review. Int J Nurs Sci.

[25] Benton JS, Cotterill S, Hawkes RE, Miles LM, French DP (2022). Changes in a digital type 2 diabetes self-management intervention during national rollout: mixed methods study of fidelity. J Med Internet Res.

[26] Glechner A, Keuchel L, Affengruber L (2018). Effects of lifestyle changes on adults with prediabetes: a systematic review and meta-analysis. Prim Care Diabetes.

[27] Wang Y, Chai X, Wang Y (2025). Effectiveness of different intervention modes in lifestyle intervention for the prevention of type 2 diabetes and the reversion to normoglycemia in adults with prediabetes: systematic review and meta-analysis of randomized controlled trials. J Med Internet Res.

[28] Higgins JPT, Altman DG, Gøtzsche PC (2011). The cochrane collaboration’s tool for assessing risk of bias in randomised trials. BMJ.

[29] Long HA, French DP, Brooks JM (2020). Optimising the value of the critical appraisal skills programme (CASP) tool for quality appraisal in qualitative evidence synthesis. Research Methods in Medicine & Health Sciences.

[30] Shea BJ, Reeves BC, Wells G (2017). AMSTAR 2: a critical appraisal tool for systematic reviews that include randomised or non-randomised studies of healthcare interventions, or both. BMJ.

[31] Hong QN, Gonzalez-Reyes A, Pluye P (2018). Improving the usefulness of a tool for appraising the quality of qualitative, quantitative and mixed methods studies, the Mixed Methods Appraisal Tool (MMAT). J Eval Clin Pract.

[32] Pluye P, Gagnon MP, Griffiths F, Johnson-Lafleur J (2009). A scoring system for appraising mixed methods research, and concomitantly appraising qualitative, quantitative and mixed methods primary studies in mixed studies reviews. Int J Nurs Stud.

[33] Rodgers M, Sowden A, Petticrew M, Arai L, Roberts H, Britten N (1995). Testing methodological guidance on the conduct of narrative synthesis in systematic reviews: effectiveness of interventions to promote smoke alarm ownership and function. Evaluation (Lond).

[34] Hussain W, Grundy J (2025). Advice for diabetes self-management by chatgpt models: challenges and recommendations. Arxiv.

[35] Sun H, Zhang K, Lan W (2023). An AI dietitian for type 2 diabetes mellitus management based on large language and image recognition models: preclinical concept validation study. J Med Internet Res.

[36] Frediani JK, Bienvenida AF, Li J, Higgins MK, Lobelo F (2020). Physical fitness and activity changes after a 24-week soccer-based adaptation of the U.S diabetes prevention program intervention in Hispanic men. Prog Cardiovasc Dis.

[37] Ruiz-Leon AM, Casas R, Castro-Barquero S (2025). Efficacy of a mobile health-based behavioral treatment for lifestyle modification in type 2 diabetes self-management: greenhabit randomized controlled trial. J Med Internet Res.

[38] Wang SCY, Nickel G, Venkatesh KP, Raza MM, Kvedar JC (2024). AI-based diabetes care: risk prediction models and implementation concerns. NPJ Digit Med.

[39] Mashatian S, Armstrong DG, Ritter A (2025). Building trustworthy generative artificial intelligence for diabetes care and limb preservation: a medical knowledge extraction case. J Diabetes Sci Technol.

[40] Alloatti F, Bosca A, Di Caro L, Pieraccini F (2021). Diabetes and conversational agents: the AIDA project case study. Discov Artif Intell.

[41] Salari R, R Niakan Kalhori S, GhaziSaeedi M, Jeddi M, Nazari M, Fatehi F (2021). Mobile-Based and cloud-based system for self-management of people with type 2 diabetes: development and usability evaluation. J Med Internet Res.

[42] Mehraeen E, Mehrtak M, Janfaza N (2022). Design and development of a mobile-based self-care application for patients with type 2 diabetes. J Diabetes Sci Technol.

[43] LeSeure P, Chin E, Zhang S (2024). A culturally sensitive mobile app (DiaFriend) to improve self-care in patients with type 2 diabetes: development study. JMIR Diabetes.

[44] Ayers JW, Poliak A, Dredze M (2023). Comparing physician and artificial intelligence chatbot responses to patient questions posted to a public social media forum. JAMA Intern Med.

[45] Nguyen TT, Sim K, Anthony To Yiu K, O’Donnell RR, Lim ST, Wang W (2021). arXiv. Designing AI-Based Conversational Agent for Diabetes Care in a Multilingual Context.

[46] Shiferaw MW, Zheng T, Winter A, Mike LA, Chan LN (2024). Assessing the accuracy and quality of artificial intelligence (AI) chatbot-generated responses in making patient-specific drug-therapy and healthcare-related decisions. BMC Med Inform Decis Mak.

[47] de Souza Ferreira E, de Aguiar Franco F, Dos Santos Lara MM (2023). The effectiveness of mobile application for monitoring diabetes mellitus and hypertension in the adult and elderly population: systematic review and meta-analysis. BMC Health Serv Res.

[48] Yeh MC, Lau W, Keady CA (2023). Evaluation of feasibility and acceptability of a web-based diabetes prevention program (DPP) for diabetes risk reduction in Chinese Americans in New York City. Front Public Health.

[49] Zhang J, Oh YJ, Lange P, Yu Z, Fukuoka Y (2020). Artificial intelligence chatbot behavior change model for designing artificial intelligence chatbots to promote physical activity and a healthy diet: viewpoint. J Med Internet Res.

[50] Mathioudakis N, Lalani B, Abusamaan MS (2025). An AI-powered lifestyle intervention vs human coaching in the diabetes prevention program. JAMA.

[51] Elhadd T, Mall R, Bashir M (2020). Artificial Intelligence (AI) based machine learning models predict glucose variability and hypoglycaemia risk in patients with type 2 diabetes on a multiple drug regimen who fast during ramadan (The PROFAST - IT Ramadan study). Diabetes Res Clin Pract.

[52] Shamanna P, Erukulapati RS, Shukla A (2024). One-year outcomes of a digital twin intervention for type 2 diabetes: a retrospective real-world study. Sci Rep.

[53] Bailey-Davis L, Wood GC, Cook A (2021). Communicating personalized risk of diabetes and offering weight reduction program choice: recruitment, participation, and outcomes. Patient Educ Couns.

[54] Brzan PP, Rotman E, Pajnkihar M, Klanjsek P (2016). Mobile applications for control and self management of diabetes: a systematic review. J Med Syst.

[55] Lim SL, Tay MHJ, Ong KW (2022). Association between mobile health app engagement and weight loss and glycemic control in adults with type 2 diabetes and prediabetes (D’LITE Study): prospective cohort study. JMIR Diabetes.

[56] Li M, Liu S, Yu B (2025). Assessing the effectiveness of digital health behavior strategies on type 2 diabetes management: systematic review and network meta-analysis. J Med Internet Res.

[57] Nanditha A, Thomson H, Susairaj P (2020). A pragmatic and scalable strategy using mobile technology to promote sustained lifestyle changes to prevent type 2 diabetes in India and the UK: a randomised controlled trial. Diabetologia.

[58] Green JB, Crowley MJ, Thirunavukkarasu S, Maruthur NM, Oldenburg B (2024). The final frontier in diabetes care: implementing research in real-world practice. Diabetes Care.

[59] Jabara M, Kose O, Perlman G (2024). Artificial intelligence-based digital biomarkers for type 2 diabetes: a review. Can J Cardiol.

[60] Nadarzynski T, Knights N, Husbands D (2024). Achieving health equity through conversational AI: a roadmap for design and implementation of inclusive chatbots in healthcare. PLOS Digit Health.

[61] Al-Hamdan R, Avery A, Al-Disi D, Sabico S, Al-Daghri NM, McCullough F (2021). Efficacy of lifestyle intervention program for Arab women with prediabetes using social media as an alternative platform of delivery. J Diabetes Investig.

[62] Bergenstal RM, Johnson M, Passi R (2019). Automated insulin dosing guidance to optimise insulin management in patients with type 2 diabetes: a multicentre, randomised controlled trial. Lancet.

[63] Xiao Y, Wang Z, Zhang L (2025). Effectiveness of digital diabetes management technology on blood glucose in patients with type 2 diabetes at home: systematic review and meta-analysis. J Med Internet Res.

[64] Röhling M, Kempf K, Banzer W (2020). Prediabetes conversion to normoglycemia Is superior adding a low-carbohydrate and energy deficit formula diet to lifestyle intervention-a 12-month subanalysis of the ACOORH trial. Nutrients.

[65] Nomura A, Noguchi M, Kometani M, Furukawa K, Yoneda T (2021). Artificial intelligence in current diabetes management and prediction. Curr Diab Rep.

[66] Lim D, Meier L, Cadwell KM, Jacob C (2025). From diabetes care to prevention: review of prediabetes apps in the DACH region. Mhealth.

[67] Abusamaan MS, Ballreich J, Dobs A (2024). Effectiveness of artificial intelligence vs. human coaching in diabetes prevention: a study protocol for a randomized controlled trial. Trials.

[68] Al‐Hamdan R, Avery A, Al‐Disi D, Sabico S, Al‐Daghri NM, McCullough F (2021). Efficacy of lifestyle intervention program for Arab women with prediabetes using social media as an alternative platform of delivery. J of Diabetes Invest.

[69] Röhling M, Kempf K, Banzer W (2020). Prediabetes conversion to normoglycemia Is superior adding a low-carbohydrate and energy deficit formula diet to lifestyle intervention-A 12-month subanalysis of the ACOORH trial. Nutrients.

[70] Wu Y, Min H, Li M (2023). Effect of artificial intelligence-based health education accurately linking system (AI-HEALS) for type 2 diabetes self-management: protocol for a mixed-methods study. BMC Public Health.

