# Supplementary material for: Digital Health Solutions for Type 2 Diabetes and Prediabetes: Systematic Review of Engagement Barriers, Facilitators, and Outcomes
Source: JMIR Diabetes. 2026 Mar 12;11:e80582. doi: 10.2196/80582 (PMC12981377; doi:10.2196/80582)
Supplement: Multimedia Appendix 1 [file diabetes-v11-e80582-s001.doc]

**Table S1.** Search Strategy

| **Step 1: Preliminary Search Keywords in Medline** | | | |
| --- | --- | --- | --- |
| type 2 diabetes, prediabetes, digital health*, artificial intelligence, machine learning, chatbot*, engagement barriers, user-centered design, self-determination theory, HbA1c reduction, adult* | | | |
| **Step 2: Keywords Used for Final Search** | | | |
| **PICO Component** | **Population** | **Intervention** | **Outcome** |
| Description | Adults aged 18–75 with type 2 diabetes or prediabetes | AI-driven/ non-AI driven digital health solutions for lifestyle management | Engagement, adherence, and clinical outcomes in diabetes self-management and prevention |
| MeSH Headings | - Diabetes Mellitus, Type 2 | - Artificial Intelligence | - Self-Management |
|  | - Prediabetic State | - Machine Learning | - Patient Compliance |
|  | - Glucose Intolerance | - Mobile Applications | - Treatment Adherence and Compliance |
|  | - Adult | - Telemedicine | - Patient Dropouts |
|  |  | - Text Messaging | - Usability Testing |
|  |  | - Wearable Electronic Devices |  |
|  |  | - Digital Health |  |
| Synonym List | - Type 2 diabet* | - AI | - Prevention |
|  | - T2DM | - Chatbot* | - Glycemic Control |
|  | - Prediabet* | - Conversational Agent* | - Weight Loss |
|  | - Adult* | - Mobile App* | - Physical Activity |
|  | - 18-75 years | - Mobile Health | - Dietary Management |
|  |  | - mHealth | - Engagement |
|  |  | - Web-based Solution* | - Adherence |
|  |  | - Telehealth | - Dropout |
|  |  | - Wearable Device* | - Attrition |
|  |  | - Lifestyle Intervention | - Barrier* |
|  |  |  | - Facilitator* |
|  |  |  | - Usability |
|  |  |  | - Acceptability |

**Table S2.** Databases Searched

| **Step 1: Preliminary Databases Searched** |
| --- |
| - Medline |
| **Step 2: Databases Searched After Refining Keywords** |
| - PubMed - Scopus - CINAHL - ScienceDirect - Google Scholar |
| - Reference lists of included studies - Handsearching of relevant journals and conference proceedings |

The search strategy employed Boolean operators to combine keywords and MeSH terms effectively, such as “(Diabetes Mellitus, Type 2 OR T2DM OR Prediabetes) AND (Artificial Intelligence OR Mobile Applications OR Wearable Devices)” to ensure comprehensive coverage. Proximity operators and truncation (e.g., “Chatbot*”) were used to capture variations in terminology across databases. Handsearching involved reviewing reference lists of included studies and key journals (e.g., Journal of Medical Internet Research, Diabetes Care).
