# Supplementary material for: Digital Health Solutions for Type 2 Diabetes and Prediabetes: Systematic Review of Engagement Barriers, Facilitators, and Outcomes
Source: JMIR Diabetes. 2026 Mar 12;11:e80582. doi: 10.2196/80582 (PMC12981377; doi:10.2196/80582)
Supplement: Multimedia Appendix 2 [file diabetes-v11-e80582-s002.doc]

**Supplementary Appendix 2: Detailed Study Characteristics and Findings**

**Table 2a: Characteristics and Findings of Included Studies**

| **Author/ Year Country** | **Purpose** | **Study type/ methodology/ sample** | **Findings related to the SLR** | **Limitations** |
| --- | --- | --- | --- | --- |
| (LeSeure et al., 2024)  USA | Develop a prototype diabetes management app for Portuguese Americans with T2DM using design thinking methodology, incorporating user-centered design and cultural sensitivity. | Mixed methods / Design thinking (ideate, prototype) / 22 participants (phase 1 interviews); phase 2 by multidisciplinary team (2 nurse educators, 1 app specialist, 2 graduate assistants). | **Barriers**: Lack of in-person user collaboration (COVID-19 restrictions), incomplete backend, English-only interface, no specialist input. **Facilitators**: Culturally tailored carbohydrate tracking, simple interface, no login, prioritized walking. **Intervention**: DiaFriend app (v1) with 5 features: blood glucose monitoring, weight tracking, carbohydrate tracking, exercise log, medication reminder, built using Flutter (v2.2). **Outcomes**: Functional front-end prototype; culturally tailored carbohydrate tracking; incomplete backend. | No real-time feedback/notifications, incomplete backend, English-only, limited user/specialist collaboration due to COVID-19.  Quality appraisal score 94.74% (MMAT) |
| (Oh et al., 2021)  USA | Evaluate AI chatbot characteristics, functions, conversational capacities, and efficacy in changing physical activity, diet, weight management, and related health outcomes. | Systematic review / PRISMA, PICOS framework / 9 studies (4 RCTs, 5 quasi-experimental), 891 participants (19-274 per study). | **Barriers**: Inconsistent outcome measures, limited reporting of engagement/satisfaction, small sample sizes in quasi-experimental studies, lack of adverse event reporting, no gray literature search, English-only studies. **Facilitators**: Personalized goal-setting, relational strategies (e.g., empathy), unconstrained chatbots for natural interaction. **Intervention**: AI chatbots (constrained/unconstrained) on platforms like Slack, KakaoTalk, Facebook Messenger, Telegram, WhatsApp, SMS, Ally app; 1-12 weeks duration. **Outcomes**: 5/7 studies showed increased physical activity; 2/4 studies showed improved diet; 1 study showed significant weight loss (1.3 kg); engagement/satisfaction reported in 7 studies, but inconsistent. | No meta-analysis due to heterogeneity, limited RCT designs, small sample sizes, English-only studies, no gray literature.  Quality appraisal score 85.71% (AMSTAR-2) |
| (Nadarzynski et al., 2024)  UK & USA | Identify activities to reduce bias in conversational AI and make their designs and implementation more equitable. | Qualitative study / Content analysis of 17 AI guidelines, framework analysis of 33 semi-structured interviews / 33 stakeholders (10 community members, 23 industry experts/healthcare professionals). | **Barriers**: Contradictory views on time/resources needed, varying participant knowledge levels, hypothetical expert opinions, heterogeneity in responses due to rapid AI advancements. **Facilitators**: Diverse stakeholder involvement, community-driven co-production, culturally competent design, clear regulatory guidance. **Intervention**: 10-stage roadmap for equitable conversational AI design and implementation (e.g., conception, co-production, safety measures, termination). **Outcomes**: Developed a 10-stage roadmap to guide equitable AI chatbot design and deployment, emphasizing inclusivity and bias reduction. | Limited applicability across diverse healthcare systems, lack of specific guidance on activities, potential irrelevance of some aspects in resource-constrained settings.  Quality appraisal score 100% (CASP) |
| (Hussain & Grundy, 2025)  Australia | Evaluate ChatGPT versions 3.5 and 4 responses to diabetes patient queries, assessing medical knowledge depth and personalized advice for diabetes self-management. | Comparative study / Adapted methodology from Sng et al., 20 qualitative unstructured diabetes queries / Evaluated by two healthcare professionals (General Practitioner, Dietitian). | **Barriers**: Inadequate personalization (generic meal plans, lack of cultural/dietary sensitivity), poor sustained engagement (no follow-up or clarification for ambiguous inputs), high risk of errors (insulin regimen misidentification, blood glucose unit errors), low risk perception (failure to emphasize safety checks), environmental constraints (Western-centric dietary bias, limited non-English language support). **Facilitators**: Improved clarity and structure in GPT-4, systematic rotation suggestions, practical tools (e.g., apps for tracking injection sites). **Intervention**: ChatGPT 3.5 and 4 responses to 20 queries covering diet, exercise, hypoglycemia/hyperglycemia, insulin storage/administration; no prompt engineering. **Outcomes**: GPT-4 showed slight improvements over GPT-3.5 in specificity, clarity, and structure, but persistent issues with personalization and accuracy (e.g., blood sugar unit errors, generic diet plans). | Simulated queries not reflective of real-world interactions, limited query scope, text-only analysis, no multimodal data.  Quality appraisal score 90% (CASP) |
| (Wang et al., 2024)  Qatar, USA / | AI-based diabetes care: risk prediction models and implementation concerns | Scoping review / PRISMA extension for Scoping Reviews, systematic search across PubMed, Scopus, IEEE-Xplore, Google Scholar / 40 longitudinal studies. | **Barriers**: Inadequate personalization (variability in risk predictors like BMI, fasting plasma glucose affecting model performance; poor demographic representation of gender, ethnicity, age in training data leading to biased predictions), environmental constraints (data quality issues and dataset variations due to regional differences in healthcare systems). **Facilitators**: Multimodal models superior due to integration of multiple data types (EHR, multi-omics, imaging), potential for digital twins using multimodal patient data. **Intervention**: Review of AI-driven risk prediction models (unimodal and multimodal) using EHR, multi-omics, and imaging data; performance measured by AUC. **Outcomes**: Multimodal models outperformed unimodal (e.g., AUC 0.96 vs. 0.586 for genomics-only), but direct comparisons challenging due to dataset variations; concerns with bias and fairness metrics. | Limited external validations (only 5 studies), few studies assessed calibration or fairness, potential biases from dataset variations, focus on classical ML models.  Quality appraisal score 90% (CASP) |
| (Sun et al., 2023)  China | Evaluate the competence of an AI-driven nutritionist program using LLMs and image recognition for T2DM dietary management. | Preclinical concept validation / Multistep process: survey of 206 T2DM patients and 26 endocrinologists, ChatGPT and GPT 4.0 tested on Chinese Registered Dietitian Exam, expert review of ChatGPT responses, development and evaluation of Dino V2-based image recognition model / 206 patients, 26 doctors, 6 expert reviewers. | **Barriers**: Low risk perception (50% of patients believed dietary management most effective, but many misidentified pork belly as higher glycemic than carrots/yams; 23% of endocrinologists made similar errors), inadequate personalization (ChatGPT’s inconsistent recommendations for Chinese-specific foods like root vegetables and dry beans due to limited training data; variable responses to same questions), environmental constraints (lack of Chinese-specific ingredients in open-source Recipe1M dataset; shortage of clinical nutritionists at 0.03 per 1000 population). **Facilitators**: Automated ingredient recognition via FMC app to reduce manual logging burden, high accuracy of Dino V2 model (F1 score 0.825), user-friendly WeChat miniprogram leveraging widespread platform access. **Intervention**: AI nutritionist program (FMC app) integrating ChatGPT for dietary advice and Dino V2 for ingredient recognition from meal images. **Outcomes**: ChatGPT and GPT 4.0 passed Chinese Registered Dietitian Exam (60.5% and 74.5% accuracy), 96.43% of ChatGPT’s ketogenic diet responses rated acceptable/excellent, Dino V2 outperformed inverse cooking model (F1 0.825 vs. 0.477). | Limited scope of tested questions, variable LLM responses, preclinical nature limits real-world adherence data, lack of Chinese-specific training data.  Quality appraisal score 100% (CASP) |
| (Wu et al., 2024)  China | Assess the potential and effectiveness of chatbots in diabetes self-management to support future chatbot development. | Systematic review and meta-analysis / Literature search in PubMed and Web of Science (until Jan 1, 2023), PRISMA-guided, 25 studies from 14 countries, including system design (n=8), pilot studies (n=8), intervention studies (n=9) / 219 participants in meta-analysis (4 pre-post trials). | **Barriers**: Poor sustained engagement (lack of voice interaction in most chatbots [76% text-based]; need for user-friendly interfaces for older adults), inadequate personalization (limited focus on psychological aspects [20% of studies]; challenges tailoring to diverse populations like older adults), environmental constraints (fewer studies in low-income countries despite high diabetes prevalence; privacy concerns due to sensitive data collection; accessibility issues for older adults due to interface design and learning barriers). **Facilitators**: Personalized advice via AI and semantic analysis, gamified approaches (e.g., virtual trophies, educational content like videos and quizzes), high user acceptance, voice interaction in some chatbots (24%), use of theoretical frameworks (e.g., Behavior Change Wheel, SDT in 40% of studies). **Intervention**: Chatbot interventions providing education and management on diet, exercise, medications, blood glucose, complications, and mental health (20% of studies). **Outcomes**: Meta-analysis showed significant reduction in HbA1c (MD 0.30, 95% CI 0.04–0.55; P=.02) but no significant weight reduction (MD 1.41, 95% CI -2.29 to 5.11; P=.46). High user acceptance and improved health status reported. | Limited high-quality evidence (only 1 RCT, mostly pre-post trials), potential publication bias (English-only studies), missing full-text articles, exclusion of gray literature, small sample sizes in pilot studies (n=10–33).  Quality appraisal score 85.71% (AMSTAR-2) |
| (Nomura et al., 2021)  Japan | Introduce AI/ML-based medical devices and prediction models for diabetes management and assess their performance. | Narrative review / Literature review with no specific methodology detailed, includes FDA-approved devices and ML prediction studies / No primary sample; secondary data from studies (e.g., 65,505 participants in Nomura’s study, 2,137,343 in Ravaut et al.). | **Barriers**: Inadequate personalization (ML models not superior to statistical models, overfitting risks reducing generalizability), environmental constraints (no AI diabetes devices approved in Japan, limited access to advanced devices in low-income countries). **Facilitators**: Large datasets (e.g., EHR, omics data), computational resources, FDA-approved devices (IDx-DR, Advisor Pro, Guardian Connect), high accuracy in specific applications (e.g., 98.5% for hypoglycemia alerts). **Intervention**: AI/ML-based devices for automatic retinal screening, clinical diagnosis support, patient self-management, and risk stratification; ML models for new-onset diabetes prediction. **Outcomes**: FDA-approved devices effective (e.g., IDx-DR for retinopathy, Advisor Pro non-inferior to specialists, Guardian Connect 98.5% accurate for hypoglycemia). ML prediction AUC 0.71–0.87, not superior to statistical models (C-index 0.74–0.94). | No superior ML performance over statistical models, overfitting risks, no AI diabetes devices in Japan, limited discussion of barriers like dropout rates or engagement.  Quality appraisal score 100% (CASP) |
| (Elhadd et al., 2020)  Qatar | Develop a machine-based algorithm to predict hyperglycaemic and hypoglycaemic excursions in T2DM patients on multiple therapies fasting during Ramadan. | Prospective observational cohort sub-study / 13 patients (10 males, 3 females) with T2DM on ≥3 anti-diabetic medications, using Fitbit-2 and Freestyle Libre for 4 weeks (2 weeks pre-Ramadan, 2 weeks during Ramadan), 5 ML techniques (Linear Regression, Random Forest, SVM, XGBoost, Deep Learning), 19,540 samples, 80% training, 20% test, 5-fold cross-validation / Sample: median age 51 years, BMI 33.2 kg/m², HbA1c 7.3%. | **Barriers**: Inadequate personalization (poor hypoglycaemia prediction [27.9% accuracy]), environmental constraints (small sample size limiting hypoglycaemia event capture). **Facilitators**: Large datasets from CGM and Fitbit, clinical data (BMI, HbA1c, age, gender, SU use), time features (Ramadan vs non-Ramadan, hour/day), dose adjustments pre-Ramadan, patient education. **Intervention**: ML models (XGBoost optimal) using physical activity, EHR data, and time features to predict glucose levels; dose reduction (SU by 50%, basal insulin by 25%) pre-Ramadan. **Outcomes**: XGBoost model: R² 0.837, MAE 17.47; accurate for normal glucose (95.2%, 2584/2715) and hyperglycaemia (82.6%, 852/1031), poor for hypoglycaemia (27.9%, 48/172). | Small sample size (n=13), limited hypoglycaemia events, no adjustment of CGM data pre-Ramadan for medication changes.  Quality appraisal score 90.91% (CASP) |
| (Zhang et al., 2020)  United States | Present a literature review of chatbot use in promoting physical activity and a healthy diet, propose an AI chatbot behavior change model, and discuss ethical principles. | Literature review and theoretical framework development / Rapid review of 4 databases (PubMed, EMBASE, Web of Science, ACM Digital Library) on August 24, 2020, identifying 108 articles, 7 relevant studies included (6 unique chatbots); no primary data collection. | **Barriers**: Dropout rates (e.g., 30% stopped using Ally app), poor sustained engagement, inadequate personalization, limited reporting on theoretical frameworks and ethical considerations. **Facilitators**: Use of behavior change theories (e.g., motivational interviewing, social cognitive theory), personalized messaging, real-time data from sensors (accelerometers, GPS), just-in-time adaptive interventions (JTAIs). **Intervention**: Proposed AI chatbot behavior change model with 4 components: (1) designing chatbot characteristics and user background, (2) building relational capacity, (3) building persuasive conversational capacity, (4) evaluating mechanisms and outcomes. **Outcomes**: Preliminary evidence from reviewed studies: Ally increased step-goal achievement with cash incentives; Healthy Lifestyle Coaching chatbot increased physical activity; Tess supported weight management; Paola improved diet and weight loss; CoachAI effective with high engagement; Reflection Companion increased motivation and physical activity. | Lack of systematic reporting on chatbot development, theoretical mechanisms, and ethical considerations; limited RCTs (only 2 of 7 studies); small sample sizes in some studies.  Quality appraisal score 90.91% (CASP) |
| (Mathioudakis et al., 2025)  United States | To determine whether referral to an exclusively artificial intelligence (AI)–led lifestyle intervention based on the Diabetes Prevention Program (DPP) is noninferior to referral to a human-led DPP in achieving recommended thresholds for weight loss, hemoglobin A1c (HbA1c) reduction, and weekly physical activity among adults with prediabetes and overweight or obesity. | Phase 3, parallel-group, pragmatic, noninferiority randomized clinical trial / Intention-to-treat analysis | **Barriers:** Lower initiation in human-led DPP (82.7% vs 93.4% in AI-led); lower completion (50.3% vs 63.9%); prohibited medications in 13 participants; site differences (AI-led less effective in Baltimore, better in Reading); older age and lower BMI associated with poorer AI-led performance.  **Facilitators:** Higher initiation (93.4% vs 82.7%, P=.001) and completion (63.9% vs 50.3%, P=.008) in AI-led; fully automated asynchronous delivery; reinforcement learning algorithm personalizing prompts via active (weight, meals) and passive (geolocation, accelerometry) data; no human coach required.  **Intervention:** AI-led DPP (Sweetch Health app + Bluetooth scale): personalized push notifications, nutrition education, gamification, meal photo detection, 12 months; vs human-led DPP (4 CDC-recognized programs, PreventT2 curriculum, remote video sessions, 16 weekly core + maintenance).  **Outcomes:** Primary composite (HbA1c <6.5% throughout + ≥5% weight loss OR ≥4% weight loss + ≥150 min/wk MVPA OR ≥0.2% HbA1c reduction) achieved by 58/183 (31.7%) AI-led vs 59/185 (31.9%) human-led; risk difference −0.2% (1-sided 95% CI −8.2%), meeting −15% noninferiority; consistent across components and sensitivity analyses; no difference in incident HbA1c ≥6.5% (4.4% vs 3.8%, P=.78); higher engagement-outcome correlation in both (completers: 37% vs 35% achieved outcome). | Pragmatic design (referral only, no study team delivery); potential intragroup correlation in human-led (shared cohorts); engagement definitions not equivalent (asynchronous vs synchronous); prohibited medications; site/age/BMI subgroup heterogeneity; no cost-effectiveness or long-term data; updates to AI app during trial.  100% (CONSORT checklist for noninferiority RCTs) |
| (Jabara et al., 2024)  Canada | Provide an overview of AI-driven digital biomarkers for T2DMM screening, complication diagnosis, and management. | Narrative review / Synthesis of studies using AI and digital health technologies / No primary data collection, sample not applicable. | **Barriers**: Data privacy, algorithm interpretability, regulatory considerations, small sample sizes, lack of external validation, limited generalizability across demographics, inconsistent preprocessing descriptions, cybersecurity risks, integration into legacy systems, costs. **Facilitators**: Multisensor devices, wearable technology, integration with EHRs, cloud-based platforms (e.g., Microsoft Azure, AWS, Google Cloud), HL7 standards for interoperability, decreasing costs of personal devices, regulatory frameworks (e.g., FDA, Health Canada). **Intervention**: AI-driven digital biomarkers using sensors (ECG, PPG, ACC, EDA, SKT), ML models (e.g., DNN, CNN, XGBoost, RF) for screening, complication prediction, and glucose monitoring. **Outcomes**: Improved screening, complication detection (e.g., retinopathy, neuropathy, nephropathy), blood glucose and HbA1c estimation; specific outcomes include IDx-DR (sensitivity 87.2%, specificity 90.7%), EyeArt (sensitivity 95.5%, specificity 85.0%), PPG-based glucose estimation (MAE 1.76 mg/dL). | Small sample sizes, lack of diverse datasets, limited external validation, potential overfitting in complex models, regulatory approval challenges, cybersecurity concerns, inconsistent data preprocessing.  Quality appraisal score 75% (CASP) |
| (Shiferaw et al., 2024)  United States | Assess the consistency, quality, and accuracy of ChatGPT v3.5 responses to healthcare-related inquiries. | Experimental study / 18 open-ended questions (6 each for "what," "why," "how") in anticoagulation, diabetes, and CKD, submitted in duplicate by 2 users, scored by 5 investigators using a 4-point scale, Delphi method for ≥80% consensus, verified with professional references / Sample: not applicable, no human subjects. | **Barriers**: Inconsistent responses between users, inaccurate calculations, unit assumptions, AI hallucinations, unreliable references, lack of personalization in clinical scenarios. **Facilitators**: Real-world question phrasing, duplicate experiment design, Delphi method for scoring consensus, use of credible references for verification. **Intervention**: Submission of 18 questions to ChatGPT v3.5 via 2 computers using Chrome and Safari browsers. **Outcomes**: ChatGPT more accurate for "what" questions (8/12 scored 3), less reliable for "why" (3/12 scored 0) and "how" (2/12 scored 0); errors in calculations (e.g., vancomycin dosing), unit assumptions, protocol misuse (e.g., warfarin dose adjustment), and non-existent references; total scores: 27 ("what"), 22 ("why"), 12 ("how"). | Limited to 3 clinical areas, used free ChatGPT v3.5, potential for different performance in newer versions, small question set (18).  Quality appraisal score 80% (CASP) |
| (Mashatian et al., 2024)  United States | Assess a retriever-augmented generation (RAG) model's ability to provide accurate, user-friendly answers on diabetes and diabetic foot care for laypersons with an eighth-grade literacy level. | Experimental study / RAG model using Python, OpenAI API, 200+ documents from CDC, ADA, AHA, IWGDF, 58 keywords, 3 rounds of literature search and validation with 175-199 questions, evaluated by 5 medical experts / Sample: not applicable, no human subjects. | **Barriers**: Information bias, residual knowledge gaps, lack of user-specific personalization, potential algorithmic bias due to limited demographic data. **Facilitators**: National Standards for Diabetes Self-Management Education, reputable sources (CDC, ADA), few-shot learning, expert validation, patient-centered resources, web-based interface. **Intervention**: RAG model with zero-shot and few-shot learning, using vetted content to answer diabetes and DFU-related questions. **Outcomes**: 98% accuracy, recall, F1-score; 99% specificity, precision after 3 validation rounds; 94% correct answers in round 3; resolved factuality and comprehension errors with few-shot learning. | Residual knowledge gaps, lack of user-specific data, potential information bias, limited to English, requires further reinforcement learning.  Quality appraisal score 80% (CASP) |
| (Ayers et al., 2023)  United States | Evaluate the quality and empathy of ChatGPT responses to patient questions compared to physician responses. | Cross-sectional study / 195 questions from Reddit’s r/AskDocs, ChatGPT v3.5 responses generated, evaluated in triplicate by licensed healthcare professionals / Sample: 195 question-response pairs, no human subjects. | **Barriers**: AI-specific (potential for fabricated information, lack of personalization due to no patient history integration), cultural/language (English-only responses), environmental constraints (HIPAA non-compliance limits clinical use). **Facilitators**: Chatbot’s longer responses (211 vs. 52 words), high-quality and empathetic tone, potential to draft responses for clinician review. **Intervention**: ChatGPT v3.5 responses to patient questions compared with physician responses. **Outcomes**: Chatbot responses preferred in 78.6% of evaluations, rated higher quality (78.5% vs. 22.1% good/very good, 3.6 times higher prevalence) and empathy (45.1% vs. 4.6% empathetic/very empathetic, 9.8 times higher prevalence). | Non-clinical setting, lack of personalization, unvalidated quality/empathy metrics, coauthor evaluators, no specific accuracy assessment, longer chatbot responses may bias empathy perception.  Quality appraisal score 80% (CASP) |
| (Mehraeen et al., 2022)  Iran | Design, develop, and evaluate a mobile-based self-care application for T2DMM patients. | Applied developmental study / Content analysis of 32 diabetes apps, checklist of features, Java-based app development on Android, no user evaluation / Sample: not applicable, no human subjects. | **Barriers**: Socioeconomic (older patients’ unfamiliarity with technology), physical constraints (morbidities like diminished eyesight, arthritis), cultural/language (Iranian-specific localization), time-consuming feature selection. **Facilitators**: Android platform popularity in Iran, evidence-based features, integration of multiple functionalities, Volley library for network operations, support for PHR and data sharing. **Intervention**: T2DMM self-care mobile app with features: text messaging, blood glucose monitoring, insulin dose suggestions, educational messaging, exercise suggestions, alerts, medication reminders, weight/BP monitoring, diet, lifestyle, PHR, metabolic management, pedometer, reporting. **Outcomes**: App developed with 18 features, localized for Iranian patients, supports data sharing with providers, no user evaluation reported. | Time-consuming review of existing apps, no user testing, limited to Android, no evaluation of effectiveness.  Quality appraisal score 94.74% (MMAT) |
| (Nguyen et al., 2021)  Singapore | Propose design principles for AI-driven conversational agents (CAs) in diabetes care and develop AMANDA, a multilingual CA with Singaporean accent. | Design Science Research (DSR) / Literature review, 3 design principles, development of AMANDA, evaluated by 20 native judges (audio quality) and 20 nurses/clinicians (usability) / Sample: no human subjects for development, 40 evaluators. | **Barriers**: Cultural/language (limited non-English CAs, linguistic insecurity in Singapore), AI-specific (constrained input, unnatural vocal output, miscommunication risks), inadequate personalization (lack of fail-safe features). **Facilitators**: Rasa NLU, Tacotron 2 with bidirectional decoder, Singaporean accent TTS, NUH-approved knowledge base, fail-safe features (input clarification, expert referral), multilingual support (English, Simplified Chinese). **Intervention**: AMANDA: AI-driven CA for diabetes care, supports text/audio input/output, multilingual, Singaporean-accented TTS. **Outcomes**: MOS: 4.07 (naturalness), 3.98 (accent uniqueness), 3.88 (clarity); SUS: 80.625 (high usability); 70% of evaluators gave SUS ≥80. | Small IMDA dataset (8 hours), limited to English and Simplified Chinese, no real-world patient testing, need for improved button usage indication, lack of TTS disable option.  Quality appraisal score 90% (CASP) |
| (Alloatti et al., 2021)  Italy | Develop and evaluate AIDA, a conversational agent (CA) for therapeutic education in diabetes care, comprising AIDA Chatbot (text-based) and AIDA Cookbot (voice-based). | Case study / Human-computer interaction workshops with 8 diabetologists and 8 patients, co-design with scientific board, deployment on Telegram, website, and Alexa, usage data analysis / Sample: 16 workshop participants, ~4000 unique users in Nov-Dec 2020. | **Barriers**: Cultural/language (Italian-only, limited linguistic diversity), inadequate personalization (no user data collection for privacy), AI-specific (91% intent recognition, 9% misclassification), environmental constraints (older patients’ discomfort with digital tools). **Facilitators**: Scientifically validated KB, empathetic tone, multi-platform access (Telegram, website, Alexa), BERT-based NLU with rule-based fallback, recipe guidance for diabetic diets. **Intervention**: AIDA Chatbot (170 Q&A on diagnosis, lifestyle, diet, prevention) and AIDA Cookbot (61 recipes, diet Q&A) for diabetes education. **Outcomes**: ~4000 unique users, 91% correct intent recognition, diet queries dominant (50% of Chatbot, 61 recipes in Cookbot), 133 unknown queries for future KB expansion. | Italian-only, no personalization, no pass-to-human feature, limited to 170 questions and 61 recipes, no direct patient outcome evaluation.  Quality appraisal score 100% (CASP) |
| (Bergenstal et al., 2019)  China | To determine whether the combination of the d-Nav Insulin Guidance System (automated insulin titration) and health-care professional support is superior to health-care professional support alone in reducing HbA1c in patients with type 2 diabetes on insulin. | Multicentre, open-label, randomised controlled trial / 1:1 block randomization within site; intention-to-treat analysis; sample size 181. | **Barriers:** Limited clinician time for frequent titration; underdosing common (most patients <0.5 U/kg/day); open-label design; short 6-month duration; small number with frequent hypoglycemia (n=3 total). **Facilitators:** d-Nav automates dose-by-dose adjustment using glucose patterns; no clinician titration needed; free supplies; structured follow-up (7 contacts); scalable without increasing clinician workload. **Intervention:** d-Nav device (Hygieia): handheld glucose meter with algorithm providing next insulin dose before each injection; analyzes patterns, adjusts every 1–7 days to prevent hypoglycemia; vs control (standard glucose meter + clinician support). Regimens: basal (1x/day), biphasic (2x/day), basal-bolus fixed/carb-counting (4x/day). **Outcomes:** Mean HbA1c reduction 1.0% (SD 1.0) intervention vs 0.3% (SD 0.9) control (p<0.0001); ≥0.5% reduction: 69% vs 36%; ≥1.0% reduction: 48% vs 19%; HbA1c <7%: 26% vs 11%; <8%: 69% vs 42%; >9%: 9% vs 23%. Hypoglycemia frequency identical (0.29 events/month both groups, p=0.96); no severe events difference. | Open-label (unmasked); short duration (6 months); limited to specialty clinics; no cost-effectiveness data; small hypoglycemia cohort; no long-term safety; possible clinician override bias; free supplies may improve adherence vs real-world, 96% (CONSORT checklist for RCTs) |
| (Al-Hamdan et al., 2021)  Saudi Arabia | Investigate the effectiveness of different educational programs (group, social media, standard care) for Saudi women with prediabetes. | Cluster-randomized, multi-intervention study / 120 Saudi women with prediabetes (40 GEP, 43 WEP, 37 CG), 6-month intervention / Sample: 253 eligible, 120 completed. | **Barriers**: Dropout rates (high initial non-response), cultural/language (Arabic-only, cultural restrictions on women’s activity), socioeconomic (limited gym access, unemployment), poor sustained engagement (no physical activity monitoring). **Facilitators**: Structured GEP sessions, WhatsApp for WEP, Al-Nahdi app, free gym access (WEP), Arabic content, certified educators. **Intervention**: GEP: 6 bimonthly in-person lifestyle sessions; WEP: same content via WhatsApp, Al-Nahdi app; CG: standard care with pamphlets. **Outcomes**: HbA1c reduced in all groups (GEP: 6.0 to 5.5, WEP: 6.0 to 5.3, CG: 6.0 to 5.7, P<0.001); GEP superior in weight loss (P=0.003), triglycerides (P<0.001), LDL-C (P=0.001), energy intake (P=0.005). | Baseline group differences, short duration, women-only, no physical activity monitoring, potential bias from unadjusted confounders.  Quality appraisal score 77.78% (CASP) |
| (Brzan et al., 2016)  Slovenia | Identify and evaluate free mobile apps for diabetes self-management based on key features (blood glucose, medication, nutrition, physical exercise, body weight). | Systematic review / PRISMA, searched Google Play, App Store, Windows Phone Store, 65 apps tested by 3 experts / Sample: 956 apps screened, 9 included. | **Barriers**: Inadequate personalization (no type-specific customization), cultural/language (English-only apps), poor sustained engagement (time-consuming data entry), socioeconomic (low health literacy, smartphone inexperience), functionality issues (56/65 apps failed). **Facilitators**: Data sharing/export, reminders, food databases, barcode scanning (I2), voice recognition (A1), community features (A3, A5, I2, W1). **Intervention**: Review of 65 free apps for diabetes self-management, 9 met all criteria (A1, A2, A3, A4, A5, I1, I2, I3, W1). **Outcomes**: 9 apps supported all key features; A1 had voice recognition and smartwatch support, I2 had barcode scanning, only 3 (A2, A3, I2) included education. | Only 3 reviewers, no real-user testing, English-only apps, free apps only, potential missed apps from non-store sources.  Quality appraisal score 87.50% (CASP) |
| (Salari et al., 2021)  Iran | Develop and evaluate a theory-based mobile and cloud-based app for T2DMM self-management and remote monitoring. | Developmental study with usability evaluation / Literature review, expert survey (21 experts), usability testing with UEQ / Sample: 14 T2DMM patients, 7 health care providers. | **Barriers**: Cultural/language (Persian-only), inadequate personalization (limited by TTM and Kreuter algorithm), poor sustained engagement (short 10-day trial), socioeconomic (smartphone literacy required), technical resource constraints. **Facilitators**: TTM-based behavioral model, Kreuter algorithm for tailored messaging, Persian food database, multi-module design (logbook, analysis, overview, education), cloud-based provider portal. **Intervention**: Mobile app with 5 modules (log-in, logbook, analysis, overview, education) for tracking BG, diet, PA, medication; cloud portal for providers; TTM-based customized messages. **Outcomes**: UEQ results: high perspicuity (patients: 2.35, providers: 2.45), novelty (2.30, 2.05), low attractiveness (1.56, 1.53); overall user satisfaction. | Small sample size (14 patients, 7 providers), short 10-day evaluation, no clinical outcome assessment, simple design reduced attractiveness.  Quality appraisal score 100% (CASP) |
| (de Souza Ferreira et al., 2023)  Brazil | Analyze the effectiveness of mobile health apps for monitoring AH and/or DM in adults and elderly. | Systematic review and meta-analysis / PRISMA, searched 5 databases, 26 studies (17 in meta-analysis), 5,288 participants from 14 countries / Sample: 1,478 for DM meta-analysis (723 control, 755 intervention). | **Barriers**: Dropout rates (not quantified), poor sustained engagement, inadequate personalization, socioeconomic (smartphone access), cultural/language (mostly Asia/Europe studies). **Facilitators**: Mobile app accessibility, complementary to usual care, educational content, behavioral change support, adherence tracking. **Intervention**: Mobile apps for monitoring AH/DM, focusing on self-management, adherence, and clinical parameters. **Outcomes**: HbA1c reduced by 0.39% (CI 0.24-0.54), improved adherence, diet, PA, weight; BP data insufficient for meta-analysis. | Limited geographic diversity (mostly Asia/Europe), no BP meta-analysis, missing fasting glucose data, some non-double-blind studies.  Quality appraisal score 85.71% (CASP) |
| (Green et al., 2024)  USA, Australia | Enhance clinical implementation of guideline-based T2DM prevention and management strategies. | Narrative review / Case studies (K-DPP, ACDC, COORDINATE-Diabetes), literature synthesis / Sample: K-DPP (1,007 participants), ACDC (755 participants), COORDINATE-Diabetes (not specified). | **Barriers**: Poor sustained engagement (low NDPP participation), socioeconomic (resource constraints, access issues), cultural/language (limited diversity in studies), inadequate personalization (generic care models), environmental constraints (rural access). **Facilitators**: Community-based approaches, telehealth infrastructure, stakeholder engagement, behavioral models (Health Action Process Approach, TTM), AI screening (retinopathy). **Intervention**: K-DPP: peer-led lifestyle intervention; ACDC: telehealth with telemonitoring, DSMES, medication management; COORDINATE-Diabetes: guideline-based therapy promotion. **Outcomes**: K-DPP: 34% T2DM incidence reduction, cost-effective; ACDC: -1.6% HbA1c reduction; COORDINATE-Diabetes: increased guideline-based therapy use. | Limited real-world implementation data, modest NDPP reach, variable telehealth reimbursement, focus on high-income settings.  Quality appraisal score 87.50% (CASP) |
| (Nayak et al., 2023)  USA | Examine if a voice-based conversational AI (VBAI) can help T2DM patients titrate basal insulin at home for rapid glycemic control. | RCT / 32 adults with T2DM needing basal insulin adjustment, 8-week follow-up, 1:1 randomization / Sample: 16 VBAI, 16 standard care. | **Barriers**: Dropout rates (7/39 randomized did not complete), cultural/language (English-only), AI-specific (rules-based, not adaptive), socioeconomic (smartphone/smart speaker access), technical barriers. **Facilitators**: Voice-based interface, daily check-ins, clinician-approved protocols, real-time data portal, high usability (89.3% data logged). **Intervention**: VBAI: Alexa-based AI for insulin titration, daily voice prompts; Standard care: clinician-led titration, online log with reminders. **Outcomes**: VBAI: faster optimal insulin dose (15 vs >56 days, P=0.006), better adherence (82.9% vs 50.2%, P=0.01), glycemic control (81.3% vs 25%, P=0.005), FBG reduction (-45.9 vs +23.0 mg/dL, P=0.001), less distress (-1.9 vs +1.7 points, P=0.03). | Short duration (8 weeks, no HbA1c), small sample, English-only, self-reported data, no VBAI vs app comparison, FBG worsening in control group.  Quality appraisal score 100% (CASP) |
| (Bailey-Davis et al., 2021)  USA | Demonstrate increased recruitment by communicating personalized T2DM risk, risk reduction with weight loss, and offering program choice; evaluate participation, weight loss, and diabetes risk reduction. | Single-arm pilot study / Pre-post design, 328 prediabetes patients from 3 primary care clinics, 6-month intervention, 12-month follow-up / Sample: 81 enrolled, 62 analyzed. | **Barriers**: Dropout rates (35.5% non-completers), low risk perception, environmental constraints (rural setting), socioeconomic (transportation barriers), inadequate personalization (DRC limitations). **Facilitators**: Personalized DRC letter, program choice (RD, DPP, WW, MEDS+RD, MEDS+DPP), free programs, Fitbit and digital scale, patient advisory council input. **Intervention**: Personalized risk letter with 3-year T2DM risk and 5-15% weight loss reduction estimates; choice of 5 lifestyle programs (6 months). **Outcomes**: Recruitment rate 25.3% (p<0.0001 vs 10%), 65% completed ≥75% contacts, 7.3% weight loss at 6 months, 6.3% at 12 months, HbA1c (p<0.0001 at 6 months, p=0.049 at 12 months), DRC reduced (p<0.0001 at 6 months), QoL improved (p=0.048 at 6 months, p=0.024 at 12 months). | Single-arm design, small sample, rural setting, self-reported data, no control group.  Quality appraisal score 100% (CASP) |
| (Frediani et al., 2020)  USA | Evaluate feasibility of a soccer-based NDPP adaptation to improve physical fitness and activity in Hispanic men with prediabetes. | Single-arm feasibility trial / Pre-post design, 24-week intervention, objective PA measures (GPS, fitness trackers), intent-to-treat analysis / Sample: 41 Hispanic men. | **Barriers**: Dropout rates (not quantified), poor sustained engagement (decline in PA), cultural/language (Hispanic focus limits generalizability), socioeconomic (employment constraints), environmental constraints (session scheduling). **Facilitators**: Culturally tailored soccer program, bilingual coaches, social support (WhatsApp, family involvement), NDPP curriculum, competitive league format. **Intervention**: 12-week soccer conditioning (2x/week, 80-min sessions with NDPP modules), 12-week maintenance (1x/week, 40-min games, NDPP discussions). **Outcomes**: Increased VO2 max (1.9% at 12 weeks, p=0.007; 1.0% at 24 weeks, p=0.036), agility (-4.7% at 12 weeks, p=0.001; -7.2% at 24 weeks, p<0.0001), strength (handgrip +7%, sit-ups +15%, push-ups +31%, vertical jump +6% at 24 weeks, p<0.05), resilience (+0.3 at 12 weeks, p=0.02); decreased daily steps (-23%, p=0.005), light PA (-26%, p<0.0001) at 24 weeks. | No control group, small sample, limited generalizability, no T2DMM incidence data.  Quality appraisal score 100% (CASP) |
| (Lim et al., 2022)  Singapore | Investigate app engagement features and their association with weight loss and glycemic control in adults with diabetes and prediabetes. | Prospective cohort study / Intervention arm of D'LITE RCT, 171 participants, 6-month follow-up, linear mixed model analysis / Sample: 99 diabetes, 72 prediabetes. | **Barriers**: Dropout rates (11/171 lost to follow-up), cultural/language (English literacy required), socioeconomic (smartphone ownership), inadequate personalization (automated cues), potential external interventions. **Facilitators**: Culturally tailored app, automated cues, 2-way dietitian chat, local food database, step tracking, glucometer and scale provided. **Intervention**: nBuddy Diabetes app: meal logging, CAL/CHO limits, step tracking, SMBG, weight charting, dietitian chat, educational videos. **Outcomes**: Weight loss: 10.6% with ≥5 app features (P<.001), 9.1% with CHO limit >5.9 days/week (P=.001), 8.8% with healthier food choices >4.3 days/week (P=.005); HbA1c reduction in diabetes: 1.2% with complete meal log >5.1 days/week or CHO limit >5.9 days/week (vs 0.2%, P<.05). | Motivated participants, digital literacy bias, potential external health interventions, self-reported data.  Quality appraisal score 100% (CASP) |
| (Nanditha et al., 2020)  India, UK | Study whether SMS text messages can motivate and educate people with prediabetes to follow lifestyle modifications to prevent type 2 diabetes. | RCT / 2062 participants with prediabetes (HbA1c 6.0-6.4%), 1:1 randomization, 2-year follow-up, intention-to-treat analysis / Sample: 1031 control, 1031 intervention. | **Barriers**: Dropout rates (14.5% control, 14.4% intervention), low risk perception, poor sustained engagement, cultural/language (India: English/local languages; UK: English), socioeconomic (access to mobile phones), inadequate personalization (TTM-based SMS). **Facilitators**: TTM-based SMS (2-3/week), baseline lifestyle education, culturally adapted messages, flexible message timing, patient involvement in SMS design (UK). **Intervention**: Control: baseline lifestyle advice; Intervention: baseline advice plus SMS messages (2-3/week) for lifestyle modification. **Outcomes**: No significant reduction in T2DM progression (HR 0.89, 95% CI 0.74-1.07, p=0.22); no significant changes in secondary outcomes (weight, BMI, BP, lipids, PA, QoL); dietary improvements reported. | Limited SMS impact, potential reporting bias, HbA1c less sensitive to behavior change, low UK diabetes incidence, cultural differences.  Quality appraisal score 100% (CASP) |
| (Rohling et al., 2020)  Germany, Austria, UK, France | Investigate if adding a low-carbohydrate and energy deficit formula diet to a low-intensity lifestyle intervention is superior in reversing prediabetes compared to lifestyle intervention alone. | RCT / Subanalysis of ACOORH study, 141 participants with prediabetes, 1:2 randomization (CON: 45, INT: 96), 52-week follow-up, ITT analysis / Sample: BMI 27-35 kg/m², HbA1c 5.7-6.4%. | **Barriers**: Dropout rates (34% at 52 weeks), inadequate personalization (non-individualized calorie targets), poor sustained engagement, socioeconomic (access to formula diet), potential selection bias. **Facilitators**: Telemonitoring (scales, pedometers), low-carbohydrate formula diet, lifestyle manuals, quarterly counselling, 4-day diet protocols. **Intervention**: CON: lifestyle intervention (diet, physical activity advice); INT: CON + formula diet (1200 kcal/day week 1, 1300-1500 kcal/day weeks 2-26, 1 meal/day weeks 27-52). **Outcomes**: INT vs. CON: higher normoglycemia conversion (50% vs. 31%, p<0.05, NNT=5.3) at 52 weeks; greater weight loss (-5.9 vs. -2.4 kg, p<0.001) at 12 weeks; improved BMI, fat mass, HbA1c (p<0.05) at 52 weeks. | No constant food diaries, LOCF imputation, no OGTT, subanalysis selection bias, power calculation based on weight loss, not conversion rate.  Quality appraisal score 100% (CASP) |
| (Yeh et al., 2023)  USA | Assess feasibility and acceptability of a culturally and linguistically tailored web-based DPP intervention among Chinese Americans with prediabetes in New York City. | Pilot study, single-arm / Mixed-methods, 13 Chinese Americans with prediabetes, 1-year intervention, quantitative (web-based surveys, CSQ-8) and qualitative (focus groups) / Sample: HbA1c 5.7-6.4%, BMI ≥22 kg/m². | **Barriers**: Dropout rates (15% at 1 year), low online literacy, cultural/language (family-style meal tracking), poor sustained engagement (low social support group use), socioeconomic (access to technology). **Facilitators**: Culturally/linguistically tailored curriculum, private Facebook group, digital pedometer, weekly reminders, user manual, flexible online schedule. **Intervention**: 1-year web-based DPP: 16-week core (weekly modules), 6-month maintenance (monthly modules), delivered via Facebook, self-monitoring (meal photos, steps). **Outcomes**: Retention: 85% at 1 year, 92% completed ≥16/22 sessions; satisfaction: CSQ-8 score 27.2/32; weight reduction: 2.3% at month 8 (p<0.05); high acceptability, increased T2DM prevention knowledge. | Small sample size, self-reported weights, no HbA1c data, limited automation, no control group, generalizability limited.  Quality appraisal score 100% (CASP) |
| (Shamanna et al., 2024)  USA | To examine glycemic control, reduced anti-diabetic medication use, and metabolic improvements after one-year in a commercial Digital Twin (DT) Precision Treatment Program for T2D. | Retrospective observational real-world study / No randomization; intention-to-treat (completers analysis); recruited Jan–Dec 2022, follow-up to Dec 2023; approved by Medisys Clinisearch Ethical Review Board; sample size 1985 | **Barriers:** Retrospective design; no control group; 6.6% dropout; self-reported adherence; commercial program (potential selection bias); heterogeneous baseline (HbA1c 5.6–16.4%, medications 0–6); no long-term safety data beyond 1 year. **Facilitators:** AI-powered DT creates dynamic metabolic model using CGM, IoT sensors, ML; integrates precision nutrition (PPGR prediction), activity, sleep, breathing; human coaching + tech nudges; real-time personalized food/activity recommendations; no calorie restriction. **Intervention:** Digital Twin (Twin Health): whole-body digital replica; sensors (CGM, wearable, scale, BP); daily inputs (meals via photo/app, activity, sleep); AI predicts PPGR, suggests optimal food/timing; deep breathing for stress; human coaching support; 1-year duration. **Outcomes:** HbA1c ↓1.8% (SD 1.7, p<0.001); 89.0% (1650/1853) achieved HbA1c <7%; medications ↓1.5 (SD 1.3, p<0.001) from 1.9 to 0.5; weight ↓4.8 kg (SD 6.0, p<0.001); HOMA2-IR ↓0.1 (SD 1.2, p<0.001); HOMA2-B ↑21.6 (SD 47.7, p<0.001); CGM: time-in-range ↑, variability ↓; 50.7% in remission (HbA1c <6.5% off meds ≥3 months). | Retrospective, no control/comparator; selection bias (commercial enrollees); 6.6% attrition; self-selection into program; no blinding; heterogeneous population; no cost-effectiveness; short-term (1 year); potential confounding from concurrent care; reliance on self-reported data; no adverse event systematic reporting. 88% (STROBE checklist for observational studies) |
| (Lim et al., 2025)  Switzerland/USA | Examine the current landscape of mHealth apps targeting prediabetes in the DACH region (Germany, Austria, Switzerland), evaluating their value for patients and clinicians, and integration into the broader healthcare ecosystem. | Review Article / Systematic search across Google Play, App Store, DiGa directory; sociotechnical framework evaluation / 76 apps identified, 8 included (e.g., DiabTrend, Glooko, CONTOUR® DIABETES App). | **Barriers:** Limited prediabetes-specific features (e.g., generic diabetes focus), poor clinician integration (e.g., inadequate workflows/training), subjectivity in assessments, lack of evidence-based validation.  **Facilitators:** Compatibility with fitness apps/glucometers, diary/logbook functionalities, certifications (CE, FDA, ISO 13485, DiGa).  **Intervention:** Evaluation of 8 apps for patient value (e.g., education, journey support), clinician value (e.g., efficiency), ecosystem fit (e.g., interoperability, data management).  **Outcomes:** No app fully met criteria; DiabTrend/Glooko excelled in patient/clinician value; emphasis on diary features but gaps in prediabetes support and evidence. | Subjectivity in criteria (low inter-rater agreement), limited to DACH region, no real-user testing, focus on available apps only (potential missed non-store apps). Quality appraisal score 85.71% (AMSTAR-2) |
| (Xue et al., 2025) China | Evaluate the effectiveness of digital health interventions (DHIs) on glycemic control and physical activity in patients with type 2 diabetes, including subgroup analyses by intervention type. | Systematic review and meta-analysis / PRISMA, searched 5 databases (Web of Science, Embase, Scopus, Cochrane, PubMed); random/fixed-effects models / 118 RCTs, 21,662 participants. | **Barriers:** High heterogeneity (I²=69–92%), inconsistent effects on physical activity/insulin resistance, limited long-term/cost data, methodological variability. **Facilitators:** Online platforms/remote monitoring most effective for HbA1c/FBG, subgroup analyses by duration/sample size, cost savings in DHIs ($269 vs. $465). **Intervention:** DHIs (mobile apps, calls/SMS, online platforms, remote monitoring) vs. usual care; outcomes**:** HbA1c, FBG, PBG, HOMA-IR, physical activity.  **Outcomes**: Significant HbA1c reduction (MD −0.32% to −0.54%), FBG (−0.30 to −0.85), PBG (SMD −0.58); no effects on HOMA-IR/physical activity; lower costs in DHIs. | High heterogeneity partially explained by covariates, limited RCTs for some outcomes, English-only studies, no gray literature, focus on short-term effects. Quality appraisal score 85.71% (AMSTAR-2) |
| (Li et al., 2025)  China, 2025 | Assessing the Effectiveness of Digital Health Behavior Strategies on Type 2 Diabetes Management: Systematic Review and Network Meta-Analysis | Systematic review & network meta-analysis / PRISMA, PROSPERO-registered, 3-round screening, behavior change techniques framework, 52 RCTs (4,000+ adults with T2DM), NMA on 27 RCTs | **Barriers:** High heterogeneity (I²=69–92%), high dropout/bias in 40+ studies, low adherence to wearables (25%), age/digital literacy gaps.  **Facilitators:** Real-time feedback, gamification, multi-strategy combos, remote monitoring. **Intervention:** 63 strategies → 19 themes (e.g., guide, monitor, engagement, communication). Top combos: HbA1c: communication + engagement + guide + management → MD −1.04% (95% CI −1.55 to −0.54); FBG: guide + management + monitor → MD −0.96; BMI/Weight: communication + engagement + goal setting + management + support → MD −2.30 / −6.50 kg.  **Outcomes:** Multi-strategy DHIs > single; positive/mixed results in most indicators; cost-effective. | High risk of bias in primary studies, short follow-ups (≤12 months), limited PA/HOMA-IR data, English-only search, no long-term adherence or equity analysis.  85.71% (AMSTAR-2) |
| (Xiao et al., 2025)  China, 2025 | Effectiveness of Digital Diabetes Management Technology on Blood Glucose in Patients With Type 2 Diabetes at Home: Systematic Review and Meta-Analysis | Systematic review and meta-analysis / PRISMA, PROSPERO-registered, searched PubMed/Embase/Web of Science/CNKI/Wanfang/CBM/Cochrane to Dec 25, 2023, Cochrane RoB for bias, meta-analysis in RevMan 5.3 / 12 RCTs (n=1,669 adults with T2DM) | **Barriers:** Not explicitly detailed; implied via biases (e.g., dropout risks, errors in manual logging). **Facilitators:** Frequent SMBG with app uploads, HCP guidance/responsiveness, automated sharing to reduce errors.  **Intervention:** Digital SMBG (apps for home monitoring/upload, sharing with HCPs) vs. control.  **Outcomes:** HbA1c MD –0.52% (95% CI –0.63 to –0.42; P <.001); FBG MD –0.42 mmol/L (95% CI –0.65 to –0.19; P<.001); 2hPBG MD –0.64 mmol/L (95% CI –0.97 to –0.32; P<.001); BMI MD –1.55 kg/m² (95% CI –2.92 to –0.17; P=.03); improved self-management. | Limited studies (n=12), high bias in some, no long-term data, focus on home only, potential publication bias.  85.71% (AMSTAR-2) |
| (Ruiz-Leon et al., 2025)  Spain, 2025 | Efficacy of a Mobile Health–Based Behavioral Treatment for Lifestyle Modification in Type 2 Diabetes Self-Management: Greenhabit Randomized Controlled Trial | RCT / 12-week, parallel, single-blind; repeated-measures ANOVA, ANCOVA; 123 participants (50% female, mean age 58.25 years, SD 9.46) recently diagnosed with T2D; intervention (n=61) Greenhabit app + standard care; control (n=62) standard care | **Barriers:** COVID-19 withdrawals (20/123), short duration (12 weeks), single-center limiting generalizability, potential technological barriers for smartphone use.  **Facilitators:** Serious gaming with points/rewards, daily messages/challenges on lifestyle (nutrition, exercise, relaxation, mindset, social), buddy system, weekly goals, group meetings to address tech issues.  **Intervention:** Greenhabit app (mHealth) for 12 weeks, focusing on 5 elements (nutrition, exercise, relaxation, positive mindset, social environment); standard care for both groups.  **Outcomes:** Both groups reduced HbA1c (-0.4% intervention vs -0.3% control, P <.001/.001) and FPG (-5.3/-5.8 mg/dL at 6 weeks, P=.01/.01); intervention additional: SBP/DBP (-4.5/-2.4 mmHg, P=.049/.03), weight (-0.8 kg, P=.03), BMI (-0.3 kg/m², P=.03), WC (-1.0 cm, P=.046), triglycerides (-20.0 mg/dL, P=.03), HDL (+2 mg/dL, P=.049); improved positivity, social environment, work-life balance. | Small sample (n=123, larger studies needed), short-term (12 weeks), single-center, COVID-19 impact on follow-up, self-reported questionnaires potential bias, no long-term outcomes.  100% (CASP) |
